# *Porphyromonas gingivalis* induces Zbp1-mediated macrophages PANoptosis in periodonitis pathophysiology

**DOI:** 10.1038/s12276-025-01443-y

**Published:** 2025-05-01

**Authors:** Jin Wu, Zixiang Guo, Long Wang, Yue Shen, Xiang Li, Zhewei Zhang, Xiao Han, Jianlan Zhang, Kunzhan Cai, Chunbo Tang

**Affiliations:** 1 State Key Laboratory Cultivation Base of Research, Prevention and Treatment for Oral Diseases, Nanjing Medical University, Nanjing, China; 2 Department of Oral Implantology Affiliated Hospital of Stomatology, Nanjing Medical University, Nanjing, China; 3 Department of Oral and Maxillofacial Surgery Affiliated Hospital of Stomatology, Nanjing Medical University, Nanjing, China

**Keywords:** Cell death and immune response, Drug delivery

## Abstract

Periodontitis is an oral immunoinflammatory disease, and macrophages play a crucial role in its pathophysiology. However, macrophage death during antibacterial activities will exacerbate inflammation and tissue damage. *Porphyromonas gingivalis* is a major constituent of subgingival biofilm plaques in periodontitis, but the effects and precise molecular mechanisms by which it triggers macrophage death remain unknown. Here we found that *P. gingivalis* infection notably activated multiple death pathways in bone-marrow-derived macrophages, including pyroptosis, apoptosis and necrosis. Furthermore, using RNA sequencing, we identified that *P. gingivalis* infection markedly increased the expression of Z-DNA binding protein 1 (Zbp1) in bone-marrow-derived macrophages. Initially identified as an interferon-induced tumor-associated protein, Zbp1 serves as an upstream sensor that regulates cell death by activating PANoptosis. Mechanistically, *P. gingivalis* induced a mitochondrial stress response, prompting the release of mitochondrial DNA. This mitochondrial DNA then interacted with Zbp1, consequently augmenting its downstream PANoptosis signals. In addition, *P. gingivalis* stimulated macrophage Zbp1 expression through the Tlr2/4–JNK–Stat3/5 pathway, exacerbating macrophage death. Importantly, blocking the biosynthesis of endogenous Zbp1 by pharmacological delivery with microneedles improved the survival of *P. gingivalis*-infected macrophages and inhibited periodontal tissue destruction. These findings highlight Zbp1 as a potential therapeutic target for *P. gingivalis*-induced periodontitis.

## Zbp1 targeting reduces macrophage death in periodontitis

Periodontitis is a gum disease that damages the tissues and bones supporting teeth. The study explores how *Porphyromonas gingivalis* worsens this condition by affecting immune cells called macrophages. Researchers found that *P. gingivalis* triggers a process called PANoptosis, where multiple cell death pathways activate simultaneously, leading to inflammation and tissue damage. The study involved analyzing gum samples from patients with periodontitis and healthy individuals, as well as using mouse models to understand the disease’s progression. They discovered that *P. gingivalis* increases the expression of a protein called Zbp1 in macrophages, which amplifies cell death signals and worsens inflammation. Researchers concluded that targeting Zbp1 could be a potential strategy to reduce tissue damage in periodontitis. They also tested a compound, salvianolic acid B, through microneedle patches, which showed promise in reducing inflammation.

This summary was initially drafted using artificial intelligence, then revised and fact-checked by the author.

## Introduction

Periodontitis is an oral immunoinflammatory disease characterized by pathological loss of the periodontal ligament and alveolar bone, driven by intricate interactions between specific pathogens and destructive immune responses^1^. *Porphyromonas gingivalis*, an anaerobic Gram-negative rod, is a key constituent of subgingival biofilm plaques and is recognized as a primary predisposing factor in the development of chronic periodontitis (CP)^2^. In periodonitis pathophysiology, *P. gingivalis* disrupts host–microbe homeostasis, evades immune surveillance and manipulates immune signaling pathways to establish a state of chronic inflammation. Its array of virulence factors, including lipopolysaccharides (LPS), gingipains and fimbriae, directly interact with immune cells, subverting their functions and contributing to periodontal tissue destruction^3^.

Macrophages are essential components of the innate immune system and are actively recruited to infection sites during inflammatory responses. They play a pivotal role in clearing microorganisms and cellular debris through phagocytosis and cytokine release^4^. Paradoxically, macrophage death during the antibacterial response may amplify proinflammatory signaling^5^. This phenomenon can not only exacerbate immune-mediated tissue damage but also contribute importantly to periodontal tissue destruction and alveolar bone resorption. Therefore, exploring the mechanisms underlying macrophage death and its impact on periodontal pathogenesis is critical to elucidating the disease’s progression and developing targeted therapeutic strategies.

Recent studies indicate that multiple cell death modes, including pyroptosis, apoptosis and necroptosis, have been detected simultaneously in rodent periodontitis models and cellular experiments^6,7^. He et al.^8^ reported that glycolysis mediates macrophage pyroptosis in periodontal diseases, resulting in increased inflammation and bone resorption. Moreover, periodontal pathogens can induce macrophage apoptosis, enabling deeper tissue invasion and exacerbating pathology^9^. MLKL-mediated necroptosis in macrophages promotes the upregulation of TNF-α, IL-1β, IL-6, COX-2 and MMP9, contributing to alveolar bone resorption in mouse periodontitis models^10^. These findings highlighted the extensive involvement of various cell death processes in periodontitis pathophysiology.

In 2019, Malireddi et al.^11^ introduced the concept of PANoptosis, where multiple cell death pathways occur simultaneously, enormously amplifying inflammatory signals. Jiang et al. further explored the association between *P. gingivalis*-induced cell death and periodontitis progression, proposing that PANoptosis contributes to the immune evasion of *P. gingivalis* and represents a potential pathogenic mechanism in periodontal disease^12^. Z-DNA binding protein 1 (Zbp1), identified as an interferon (IFN)-induced tumor-associated protein, contains two left-handed helical nucleic acid-binding domains (Zα) and two RIP homotypic interaction motif (RHIM) domain that facilitates protein–protein interactions^13^. Zbp1 is initially acknowledged for its involvement in antiviral responses, aiming to characterize the upstream receptors that mediate the activation of PANoptosis pathways during influenza A virus infection^14^. Recent studies have expanded their role in inducing cell death during bacterial infections^15^. Although moderate Zbp1 activation contributes to the host defense against certain pathogens, evidence suggests that excessive Zbp1 induction may paradoxically exacerbate chronic inflammation^15^. For example, key necrosis-related regulatory pathways predicted by the ceRNA network indicated that the RP11-138A9.1–hsa-miR-98-5p–Zbp1 axis markedly amplified periodontal inflammation^16^. In addition, metformin was used to alleviate apical periodontitis by inhibiting Zbp1-mediated necrosis^17^. Thus, Zbp1 may contribute to tissue damage through the activation of the cell death pathway in immune regulatory processes. However, its role in *P. gingivalis*-induced periodontal inflammation is not fully understood.

In this study, we characterized the abundance of macrophages within periodontitis tissues using single-cell RNA sequencing analysis. Subsequently, we presented evidence that *P. gingivalis* infection substantial intensified Zbp1-mediated macrophage death and periodontal inflammation through interactions with mitochondrial DNA (mtDNA) and activation of the Tlr2/Tlr4–JNK–Stat3/Stat5 signaling pathways. The potential of Zbp1 as a therapeutic target for alleviating *P. gingivalis*-induced macrophage death and periodontal resorption has also been explored, suggesting a theoretical basis for novel targeted tissue regeneration strategies in periodontitis.

## Materials and methods

### Specimen collection and preparation

Subgingival plaque samples were obtained from six patients with CP and five healthy individuals during health examinations. Sterile curette scalers were used to collect subgingival plaques from the areas between the gingival margin and 3 mm below it, and the samples were promptly stored at −80 °C. The inclusion criteria for CP in this study followed the 2018 updated classification of the periodontal workshop^18^, encompassing patients who (1) had not received treatment for periodontitis in the preceding 6 months, (2) exhibited normal blood glucose levels without diabetes mellitus, (3) had a minimum of four teeth with a probing depth ≥6 mm, (4) had clinical attachment loss ≥3 mm, (5) had a bleeding on probing index ≥2 and (6) had evident bone loss observed through radiographic examination. The clinical characteristics of the patients are summarized in Supplementary Table 1.

Gingival specimens were obtained during crown lengthening or oral implant surgery from five patients with CP and five healthy individuals (Supplementary Table 2). The collected gingival tissues were fixed in 4% paraformaldehyde for 24 h, followed by dehydration, embedding and sectioning into 4-μm frozen slices. The protocols for collecting subgingival plaque samples and gingival specimens were approved by the Research Ethics Committee of Nanjing Medical University (PJ2023-089-001), and informed consent was obtained from all participants.

### 16S rRNA sequencing

16S rRNA gene amplicon sequencing and analysis were conducted by OE Biotech. Bacterial DNA was extracted from subgingival plaque samples using the DNeasy PowerSoil kit (Qiagen) following the manufacturer’s instructions. DNA concentration and integrity were assessed using NanoDrop 2000 (Thermo Fisher Scientific). Polymerase chain reaction (PCR) amplification of the V3–V4 hypervariable regions of the bacterial 16S rRNA gene was performed using universal primer pairs (343 F: 5′-TACGGRAGGCAGCAG-3′; 798 R: 5′-AGGGTATCTAATCCT-3′). Subsequent sequencing was conducted using the Illumina NovaSeq6000 platform (Illumina). Relative abundance of each operational taxonomic unit was calculated. The microbial diversity of the samples was assessed using alpha diversity indices, including abundance-based coverage estimator, observed operational taxonomic units, whole trees and Chao1 indexes.

### Mouse periodontitis model

Male wild-type (WT) C57BL/6N mice (8 weeks old) were housed under specific-pathogen-free conditions with a standard 12-h/12-h light/dark cycle. Mice were randomly divided into control and *P. gingivalis*-infected groups with five mice in each group. Antibiotics (amoxicillin and metronidazole) were administered in drinking water for 7 days to mitigate the impact of the resident oral microbiota^19,20^. After a 3-day antibiotic recovery period, silk ligatures (5–0) were placed around the maxillary second molars. Subsequently, *P. gingivalis* (ATCC 33277, 1 × 10^9^) suspended in phosphate-buffered saline (PBS, 20 μl) were administered onto the alveolar crest every other day, while PBS alone were injected in the control group. The maxillary bones were excised 14 days after the first injection and immediately fixed in 4% paraformaldehyde solution for 48 h. The maxillary jaws were subsequently decalcified with 20% ethylene diamine tetraacetic acid at 4 °C for 30 days. After decalcification, standard procedures for dehydration and paraffin embedding were used for histological analyses.

*Zbp1*^−/−^ mice were established by Cyagen Biosciences using the CRISPR–Cas9 technology. Animal experimental procedures were approved by the Laboratory Animal Care and Use Committee of Nanjing Medical University (approval no. IACUC - 1905049).

### Cell isolation and culture

The culture and differentiation of bone-marrow-derived macrophages (BMDMs) adhered to procedures detailed in a previous study^21^. In brief, femurs and tibias of 8-week-old C57BL/6N mice were aseptically extracted. Subsequently, the cells were flushed from the bone marrow cavities, followed by blood cell lysis, and cultured in DMEM supplemented with 2% fetal bovine serum (Gibco) and 1% penicillin–streptomycin (Gibco) for 4 h at 37 °C in a 5% CO_2_ atmosphere. All nonadherent cells were collected and reseeded in DMEM containing 10% fetal bovine serum, 2% penicillin–streptomycin and murine M-CSF (10 ng/ml, PeproTech), with medium renewal every 3 days. BMDMs adhering to the culture flasks were collected after 7 days and used for subsequent experiments.

### Co-culture of BMDMs and *P. gingivalis*

*P. gingivalis* (ATCC 33277) was inoculated onto anaerobic blood agar medium (10% sheep blood, 10 µg/ml hemin chloride and 1.0 µg/ml vitamin K1) and incubated under anaerobic conditions (85% nitrogen, 10% hydrogen and 5% carbon dioxide) at 37 °C for 48 h. After incubation, bacterial colonies were collected and washed with PBS. *P. gingivalis* was inoculated into BMDMs at various multiplicities of infection (MOI) and cultured for 24 h. The co-culture environment was maintained under the same conditions as those used for BMDM cultivation.

### Fluorescence in situ hybridization

A fluorescence in situ hybridization (FISH) assay was performed to identify the localization of *P. gingivalis* following the manufacturer’s instructions for the Ribo Fluorescent In Situ Hybridization Kit (RiboBio). Cell or periodontal tissue samples were treated with 0.1% Triton X-100 (Beyotime), followed by prehybridization solution incubation at 37 °C for 30 min. Subsequently, they were incubated overnight in darkness at 37 °C with hybridization solution containing Cy3-labeled FISH probe (POGI: 5′-CAATACTCGTATCGCCCGTTATTC-3′, Generay Biotech). Before sealing, the nuclei were stained with DAPI (Beyotime), and images were captured using confocal laser scanning microscopy (Zeiss).

### RNA sequencing

The differentially expressed genes (DEGs) between PBS-treated BMDMs and *P. gingivalis*-infected BMDMs (MOI of 100 for 24 h) were identified through RNA sequencing, with each group comprising four cell samples (*n* = 4). Total RNA was isolated using TRIzol reagent (Invitrogen) following the manufacturer’s protocol. The TruSeq Stranded mRNA LT Sample Prep Kit (Illumina) was used for cDNA synthesis and library construction. Subsequently, the constructed libraries underwent quality assessment using an Agilent 2100 Bioanalyzer (Agilent Technologies) and were sequenced using an Illumina HiSeq6000 sequencer after passing the quality check. Gene expression was quantified in fragments per kilobase per million reads using the limma package in R software. The threshold for identifying differentially expressed genes (DEGs) was set at log_2_ fold change (FC) ≥1.5 with *P* < 0.05.

### DNA extraction

Subcellular fractionation was performed according to the previously described method^22^. In brief, BMDMs (1 × 10^6^) were collected and centrifuged (400*g*, 3 min, 4 °C), and the pellet was subsequently resuspended in hypotonic buffer and incubated on ice for 30 min. Swollen cells were homogenized 50 times with a 26-gauge needle. Low-speed centrifugation (2,500*g*, 5 min, 4 °C) was used to pellet nuclei and cellular debris. The supernatant was transferred to a new centrifuge tube, and after high-speed centrifugation (17,000*g*, 30 min, 4 °C), the supernatant was the cytoplasmic fraction. Mitochondria were enriched in the pellet, which was then washed three times with hypotonic buffer. DNA extraction was carried out separately from the cytoplasmic and mitochondrial fractions using the Quick-gDNA MiniPrep Kit (Thermo Fisher Scientific).

Genomic DNA was extracted using the GeneJET Genomic DNA Purification Kit (Thermo Fisher Scientific). BMDMs (1 × 10^6^) were collected and centrifuged (250*g*, 5 min, 4 °C). Lysis buffer and proteinase K solution were added, and samples were incubated at 56 °C for 10 min until cells were completely lysed. Subsequently, the lysate was transferred to DNA purification columns and purified using wash buffer. Genomic DNA was obtained by eluting with elution buffer and centrifugation (8,000*g*, 1 min).

### Chromatin immunoprecipitation assay

The SimpleChIP Plus Sonication ChIP kit (CST) was used for chromatin immunoprecipitation (ChIP) assays. BMDMs (1 × 10^6^) were fixed using 1% formaldehyde and subsequently collected by scraping and lysis. Chromatin was collected and fragmented into 300-bp segments by sonication. The fragments were then subjected to immunoprecipitation using anti-Zbp1 antibody (sc-271483, Santa Cruz, 5 μg) and Protein A/G. Subsequently, the protein–DNA cross-links were reversed, and the DNA was purified. The expression of mitochondrial genes *Cyt B* and *Nd2* were assessed by PCR to validate the binding of mtDNA to Zbp1. The fragments were also subjected to immunoprecipitation using antibodies against Stat3 (#12640, CST, 1:50) and Stat5 (#94205, CST, 1:50). Subsequently, the enrichment of putative binding sites for Stat3/Stat5 in the *Zbp1* promoter was evaluated via quantitative real-time PCR (qRT–PCR). The sequences of ChIP primer are listed in Supplementary Table 3.

### Microneedle patch construction and characterization

Microneedle (MN) patch was used for the delivery of salvianolic acid B (Sal B), a compound extracted from *Salvia miltiorrhiza*, into periodontal tissues. In brief, Sal B (0.5 or 1 mg, Aladdin) was dissolved in ddH_2_O (50 μl), followed by addition of polyethyleneimine (PEI, Aladdin) solution (50 μg/ml, 50 μl) and hyaluronic acid (Aladdin) solution (50 μg/ml, 50 μl). The mixture was vortexed for 30 s, and the resulting solution was allowed to stand for 1 h. Subsequently, the complex solution was added to the polydimethylsiloxane mold with a distribution area of needles measuring 4.8 mm × 4.8 mm, and arranged in an array of 10 × 10. After this, the mold was subjected to vacuum treatment for 30 min to fill the cavities. The mold was dried in a vacuum desiccator at 25 °C for 30 min. Next, an 18% aqueous polyvinyl alcohol (PVA) solution was added to the mold, which underwent vacuum treatment for an additional 30 min. The mold was stored in a vacuum desiccator at 25 °C overnight to obtain MN patches.

A texture analyzer (Brookfield CT3) was used to assess the mechanical strength of the MN patches. The MN patches were positioned with the needle tips facing upward, and the force magnitude and deformation of the MNs were recorded under a vertical compression force applied above the MNs. To further validate the effective delivery of Sal B loaded into the MNs, patches were immersed in 1 ml of PBS for 10, 20 and 30 s. The solution was then collected, and the concentration of Sal B was measured using Agilent 1260 Infinity II LC System (Agilent) to assess the release efficiency from MN loading.

### Statistical analysis

Data are presented as mean ± standard deviation, and statistical analyses were carried out using GraphPad Prism 9 software. Statistical significance of two-group comparisons was assessed using a two-tailed unpaired Student’s *t*-test. The statistical significance of the differences between more than two groups was calculated using one-way analysis of variance with Tukey’s post-hoc test. Significance was set at *P* < 0.05.

The sequences of small interfering RNAs (siRNAs) used in this study are listed in Supplementary Table 4. For complete materials and methods, please refer to the Supplementary Information.

## Results

### *P. gingivalis* infection triggered macrophage infiltration and induced apoptosis and necrosis in periodontal tissues

**Fig. 1:**
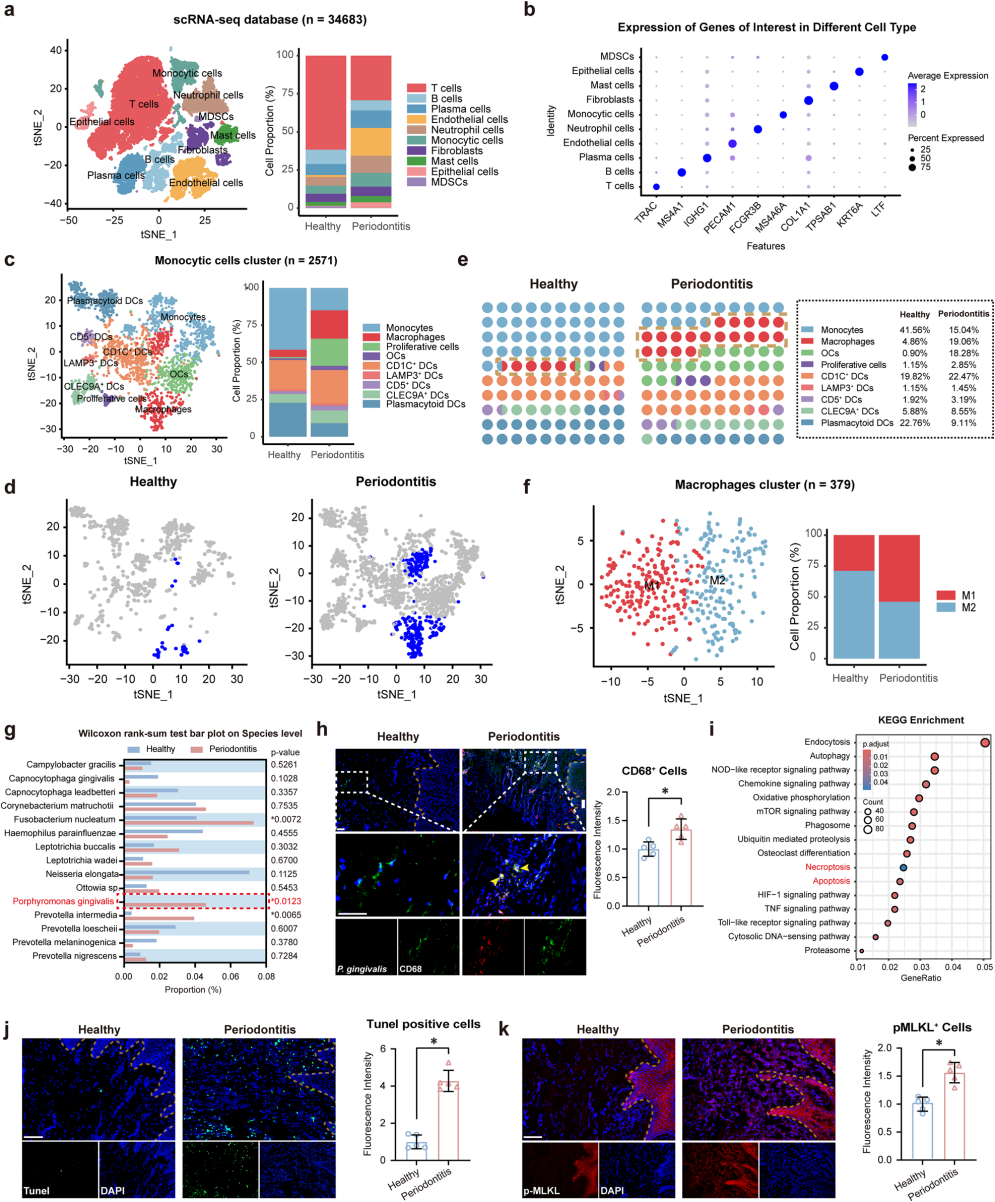
*P. gingivalis* infection triggered macrophage infiltration and induced apoptosis and necrosis in periodontal tissues. **a** t-Distributed Stochastic Neighbor Embedding (t-SNE) plot and stacked bar plots showing cell subtypes of periodontal samples from the single-cell sequencing dataset GSE171213. **b** Dot plots showing average expression levels (*Z*-scaled) and percentage of expressed cells (dot size) of marker genes in major cell types. **c** Cell subtypes of monocyte clusters. **d** t-SNE plot representing the macrophage clusters of cells identified across healthy and periodontitis. **e** Increased abundance of macrophage clusters in periodontitis samples. **f** Elevated proportion of proinflammatory M1 macrophages in periodontitis. **g** Top 10 bacterial genera in subgingival plaque. **h** FISH and IF staining showing increased *P. gingivalis* accumulation and macrophage infiltration in periodontitis tissues. *n* = 5. Scale bar, 50 μm. **i** KEGG enrichment analysis revealing abnormal increase in apoptosis and necrosis pathways in periodontitis macrophages. **j**, **k** Elevated TUNEL-positive and p-Mlkl^+^ cells in periodontitis tissues. *n* = 5. Scale bar, 50 μm. All data were derived from independent experiments. *P < 0.05.

To investigate the cell population composition and regulatory pathways in periodontitis, we analyzed periodontal samples from four healthy individuals and five patients with severe periodontitis using the single-cell sequencing dataset GSE171213 (ref. ^23^) (Fig. 1a, b). Given the pivotal role of monocyte clusters in the innate immune response, we focused on their involvement in periodontitis pathophysiology (Fig. 1c and Supplementary Fig. 1a). The analysis revealed a statistically significant increase in the abundance of macrophage clusters, particularly proinflammatory M1 macrophages, in periodontitis compared with healthy controls (Fig. 1d–f and Supplementary Fig. 1b).

Oral microbiota is vital in the development of periodontitis. Therefore, subgingival plaque samples were collected from five healthy individuals and six patients with periodontitis, and subsequently subjected to 16S rRNA sequencing. Microbiota analysis revealed increased alpha diversity in patients with periodontitis, reflecting a richer microbial community compared with healthy controls, and beta diversity analysis revealed notable differences in microbial composition between samples (Supplementary Fig. 1c, d). Figure 1g delineates the top 10 bacterial genera in abundance, with a notable enrichment of *P. gingivalis* in patients with periodontitis compared with healthy individuals. FISH and immunofluorescence (IF) staining confirmed a substantial increase in *P. gingivalis* accumulation and macrophage infiltration in gingival samples from patients with periodontitis (Fig. 1h).

Studies have shown that, during immune response, macrophage death can accelerate the release of inflammatory factors, promoting gingival destruction and periodontal bone loss^24,25^. Notably, we analyzed upregulated DEGs in macrophages from periodontitis patients compared with healthy individuals, and Kyoto Encyclopedia of Genes and Genomes (KEGG) enrichment analysis revealed a marked increase in apoptosis and necrosis pathways in periodontitis samples (Fig. 1i). IF staining confirmed the enhanced expression of proinflammatory cytokines IL-1β and TNF-α in periodontitis tissues (Supplementary Fig. 1e). Furthermore, sagittal cross-sections of gingival tissues revealed a statistically significant enrichment of terminal deoxynucleotidyl transferase dUTP nick-end labeling (TUNEL)-positive and phosphorylated mixed-lineage kinase domain-like protein (p-MLKL)^+^ cells in the periodontitis group compared with healthy individuals (Fig. 1j, k), which highlighted the substantial activation of cell death pathways in the context of periodontal inflammation.

### Increased cell apoptosis and necrosis in *P. gingivalis*-infected mouse periodontal tissues

**Fig. 2:**
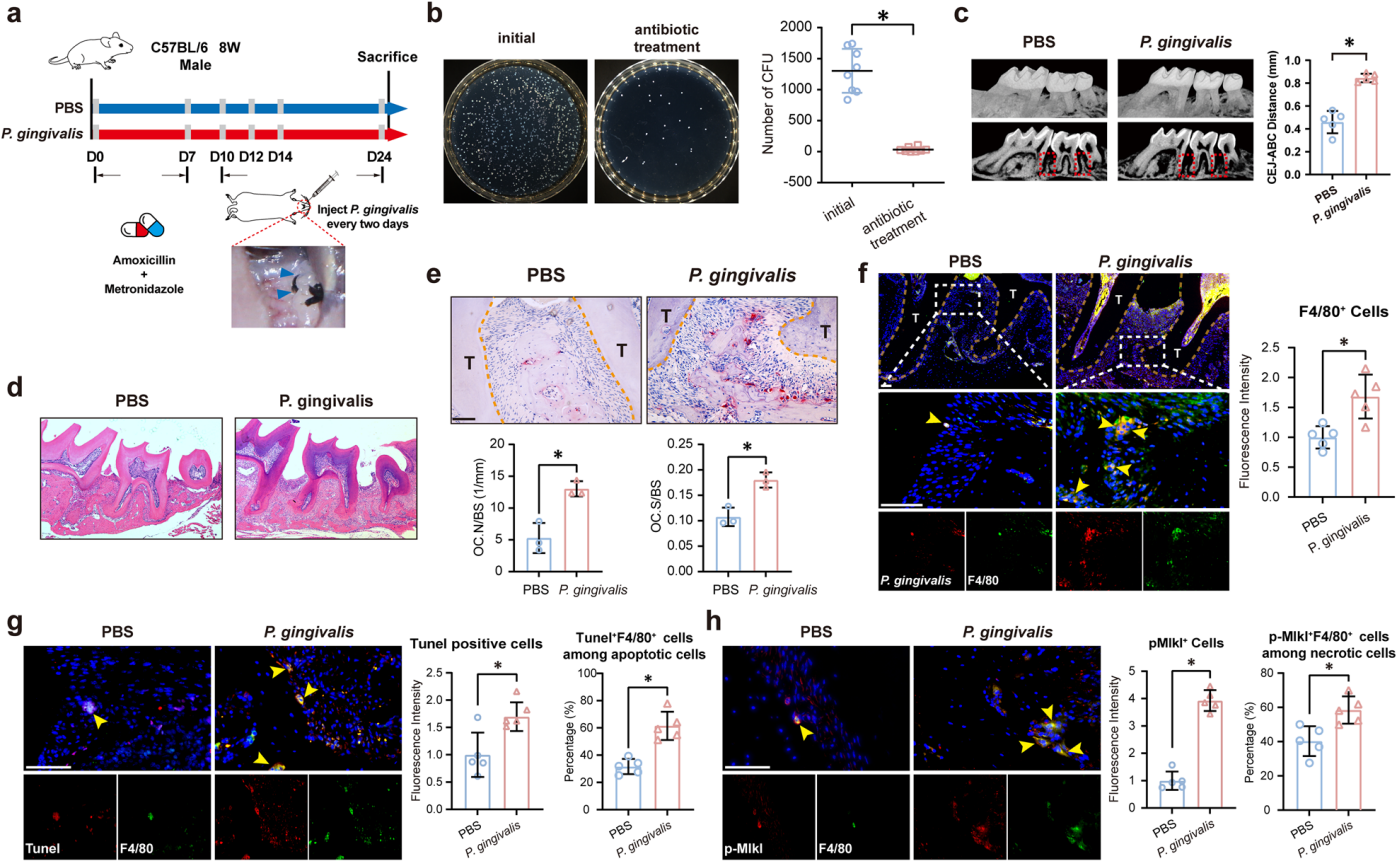
Increased cell apoptosis and necrosis in *P. gingivalis*-infected mouse periodontal tissues. **a** Establishment of a mouse periodontitis model involving the maxillary second molar. *n* = 5. **b** Reduced bacterial colonization after antibiotics treatment. *n* = 10. Representative micro-CT (**c**) and HE (**d**) staining images illustrating gingival recession and alveolar bone resorption in *P. gingivalis*-infected mice. *n* = 5. Scale bar, 50 μm. **e** Enhanced TRAP-positive osteoclasts in *P. gingivalis*-infected periodontitis tissues. *n* = 5. Scale bar, 50 μm. **f**–**h** Increased macrophage infiltration (**f**), cell apoptosis (**g**) and necrosis (**h**) in periodontal tissues from mice infected with *P. gingivalis. n* = 5. Scale bar, 50 μm. All data were derived from independent experiments. *P < 0.05.

To further explore the potential impact of sustained *P. gingivalis* infection on periodontal inflammation, we established a mouse model of periodontitis involving the maxillary second molar (Fig. 2a). Antibiotics were administered to the mice for 7 days, resulting in a statistically significant reduction in bacterial colonization in the oral cavity (Fig. 2b). Subsequently, the mice with silk ligatures were randomly divided into two groups, receiving either PBS or *P. gingivalis* suspension treatment. Micro-computed tomography (micro-CT) imaging (Fig. 2c) and hematoxylin and eosin (HE) staining (Fig. 2d) indicated a statistically significant increase in both gingival recession and alveolar bone resorption in *P. gingivalis*-infected mice compared with control mice. Tartrate resistant acid phosphatase (TRAP)-positive osteoclasts were markedly increased in the infected mice (Fig. 2e). In addition, we noted significant macrophage infiltration within the *P. gingivalis*-infected mice (Fig. 2f). Moreover, upregulated expression of proinflammatory markers Il-1β and Tnf-α was observed in *P. gingivalis*-infected periodontal tissues (Supplementary Fig. 2). Importantly, a significant increase in cell apoptosis and necrosis was detected in periodontitis tissues, along with higher relative proportions of TUNEL^+^F4/80^+^ and p-Mlkl^+^F4/80^+^ macrophages within the apoptotic and necrotic cell populations (Fig. 2g, h). These findings further indicated that *P. gingivalis* infection can exacerbate periodontitis and contribute to increased periodontal cell death.

### *P. gingivalis* infection activated Zbp1 in BMDMs

To further explore the impact of *P. gingivalis* infection on macrophages phenotype, we co-cultured mouse BMDMs with *P. gingivalis* (MOI of 100) for 24 h. The results revealed significantly higher expression of IL-1β and TNF-α, along with an increased M1 polarization in *P. gingivalis*-infected BMDMs, compared with BMDMs treated with PBS (Supplementary Fig. 3a–c). Moreover, YO-PRO-1/propidium iodide (PI) staining revealed that *P. gingivalis* stimulation increased apoptosis and necrosis in BMDMs (Supplementary Fig. 3d).

**Fig. 3:**
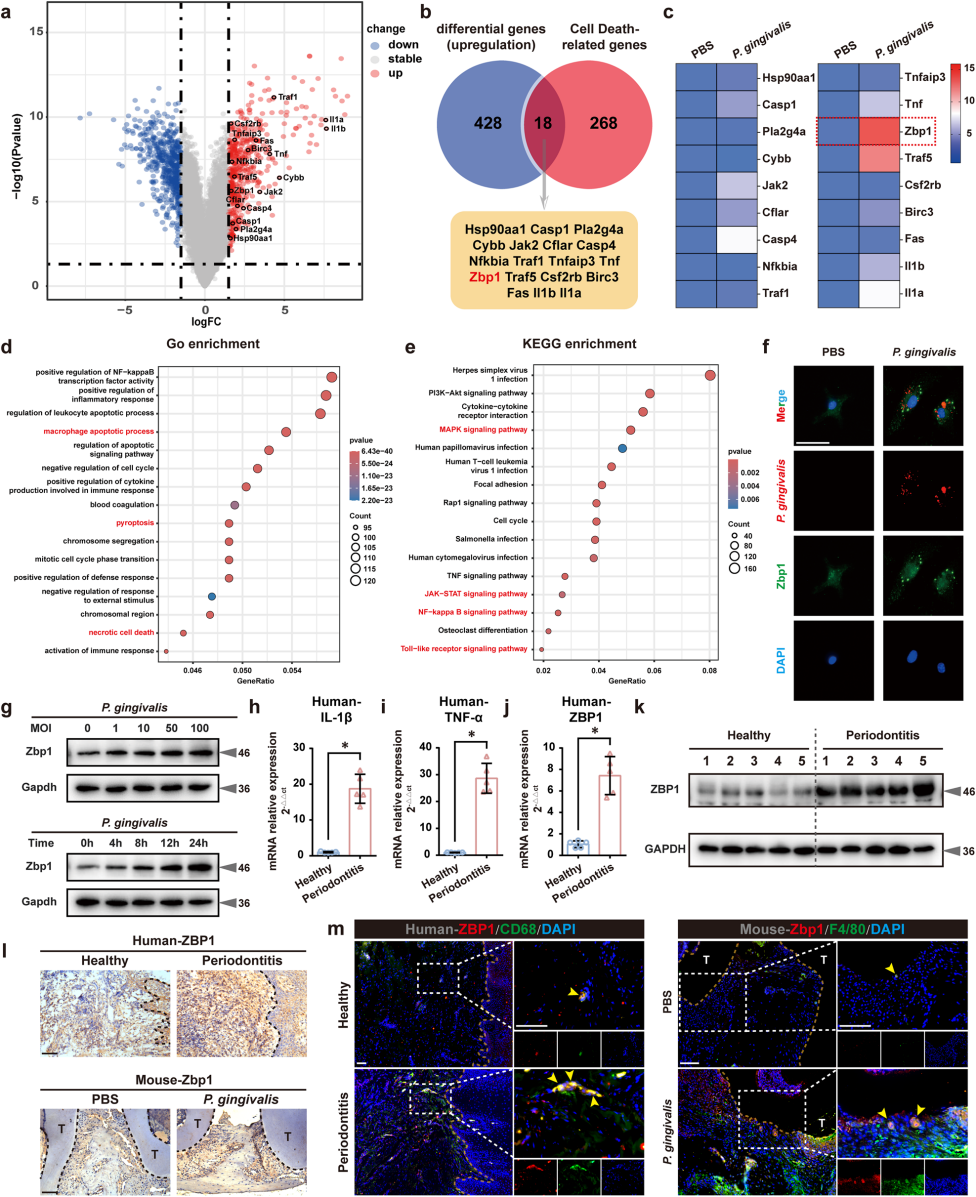
*P. gingivalis* infection activated Zbp1 in BMDMs. **a** Volcano plot of 1,068 DEGs (500 upregulated, 568 downregulated) in BMDMs treated with PBS or *P. gingivalis* (*n* = 4, log_2_FC ≥1.5 and *P* < 0.05). **b** Eighteen upregulated DEGs associated with cell death. **c** qRT–PCR showing significant upregulation of *Zbp1* expression in *P. gingivalis*-infected BMDMs. *n* = 3. GO (**d**) and KEGG (**e**) enrichment analyses revealing cell death-related pathways abnormalities. **f** FISH and IF staining showing increased Zbp1 expression in *P. gingivalis*-infected BMDMs. *n* = *3*. Scale bar, 10 μm. **g** Higher Zbp1 signals in *P. gingivalis*-induced BMDMs, displaying both dose- and time-dependent regulation. *n* = 3. **h**–**j** qRT–PCR analysis revealing elevated *IL-1β*, *TNF-α* and *ZBP1* expression in periodontitis tissues. *n* = 5. *P < 0.05. **k** Western blotting analyses of Zbp1 expression in gingival tissues from healthy and periodontitis individuals. *n* = 5. **l**, **m** IHC and IF assays confirming upregulated Zbp1 expression in CD68^+^ and F4/80^+^ macrophages. *n* = 5. Scale bar, 50 μm. All data were derived from independent experiments. *P < 0.05.

Currently, there is limited information on the mechanism by which oral microbiota induce macrophage death in periodontitis. Therefore, we subjected total RNA isolated from BMDMs treated with PBS or *P. gingivalis* to RNA sequencing (*n* = 4). A volcano plot displayed a total of 1068 identified DEGs (log_2_FC ≥1.5 and *P* < 0.05), including 500 upregulated genes and 568 downregulated genes (Fig. 3a). Immune-response-related markers were dysregulated in *P. gingivalis*-infected BMDMs (Supplementary Fig. 3e). Subsequently, we sought to uncover the intersection between upregulated DEGs and genes associated with cell death, and 18 genes were screened common to both datasets (Fig. 3b and Supplementary Fig. 3f). Importantly, qRT–PCR assays revealed the significant upregulation of *Zbp1* in *P. gingivalis*-infected BMDMs (Fig. 3c).

As a crucial innate immune sensor, Zbp1 mediates a wide range of cell death processes, leading to PANoptosome assembly^26^. Gene Ontology (GO) and KEGG enrichment analyses further indicated upregulation of pathways related to PANoptosis (including pyroptosis, apoptosis and necrosis) in *P. gingivalis*-infected macrophages compared with the control group (Fig. 3d, e). FISH and IF staining demonstrated that, after infection with *P. gingivalis*, the bacteria were engulfed by BMDMs, accompanied by increased Zbp1 expression in the infected macrophages (Fig. 3f). Moreover, western blotting assays revealed significantly higher Zbp1 signals in *P. gingivalis*-infected BMDMs than in PBS-treated cells, displaying both dose- and time-dependent regulation (Fig. 3g). Analyses of gingival tissues from healthy and periodontitis individuals demonstrated a statistically significant increase in the expressions of *IL-1β*, *TNF-α* and *ZBP*1 in infected tissues (Fig. 3h–k). Immunohistochemistry (IHC) and IF assays of human and mouse periodontal tissues further confirmed the upregulation of Zbp1 expression, particularly in CD68^+^ and F4/80^+^ macrophages (Fig. 3l, m). In summary, these data support the finding that *P. gingivalis* infection triggers Zbp1 expression in macrophages.

### *P. gingivalis* infection amplified Zbp1-mediated PANoptosis signaling

**Fig. 4:**
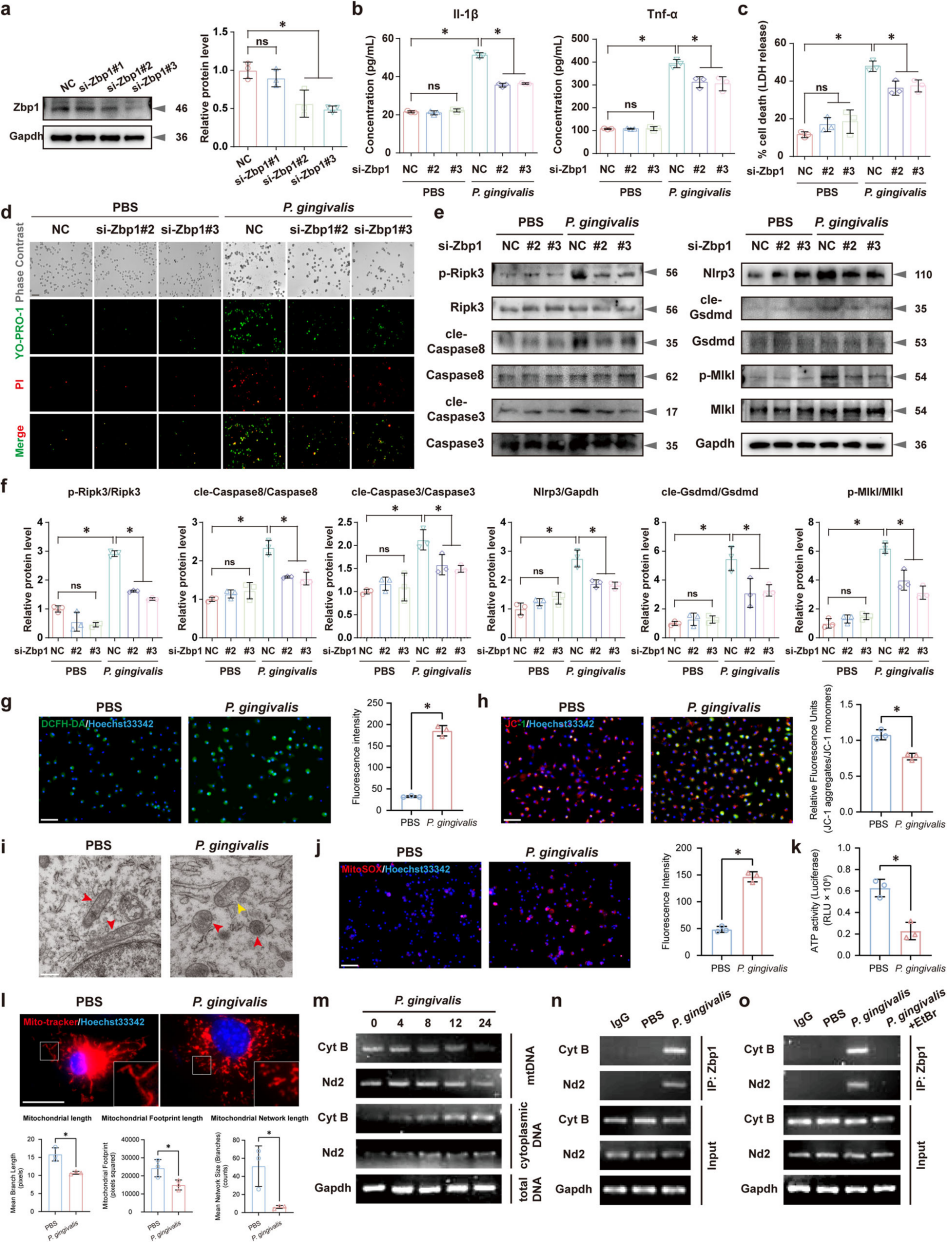
Targeted deletion of Zbp1 in BMDMs protected against PANoptosis induced by *P. gingivalis* infection. **a** *Zbp1* knockdown using *Zbp1*-specific siRNAs. *n* = 3. ELISA assay showing suppressed IL-1β, TNF-α (**b**) and LDH (**c**) release upon inhibition. *n* = 3. **d** YO-PRO-1/PI staining showing decreased apoptosis and necrosis with *Zbp1* siRNA treatment. *n* = 3. Scale bar, 50 μm. Western blotting (**e**) and quantitative analysis (**f**) demonstrated decreased p-Ripk3, cleaved Caspase8, cleaved Caspase3, Nlrp3, cleaved Gsdmd and p-Mlkl levels after *Zbp1* knockdown. *n* = 3. **g** Increased ROS levels in BMDMs upon *P. gingivalis* infection. *n* = 3. Scale bar, 50 μm. **h** JC-1 staining showing reduced Δ*ψ*_M_ after *P. gingivalis* infection. *n* = 3. Scale bar, 50 μm. **i** Swollen mitochondrial morphology in *P. gingivalis*-stimulated BMDMs. Scale bar, 500 nm. **j**, **k** Elevated mtROS levels and impaired ATP generation in *P. gingivalis*-infected BMDMs. *n* = 3. Scale bar, 50 μm. **l** Fluorescence of MitoTracker revealing increased mitochondrial fragmentation upon *P. gingivalis* stimulation. *n* = 3. Scale bar, 10 μm. **m** PCR assays showing significant cytoplasmic expression of mitochondrial genes *Cyt B* and *Nd2* after *P. gingivalis* infection. *n* = 3. **n** Interactions between mtDNA and Zbp1 identified using a ChIP experiment. *n* = 3. **o** Undetectable *Cyt B* and *Nd2* in Zbp1-bound immunoprecipitates after EtBr treatment (10 μg/ml). *n* = 3. All data were derived from independent experiments. ns, no significance, *P < 0.05.

To elucidate the regulatory role of Zbp1 in *P. gingivalis*-mediated macrophage PANoptosis, *Zbp1*-specific siRNAs were used (Fig. 4a). The enzyme-linked immunosorbent assay (ELISA) revealed a significant reduction in the expression levels of IL-1β and TNF-α in the supernatants of *P. gingivalis*-infected macrophages upon Zbp1 inhibition (Fig. 4b), accompanied with a decrease in lactate dehydrogenase (LDH) release (Fig. 4c). Moreover, YO-PRO-1/PI staining confirmed a significant increase in apoptosis and necrosis upon *P. gingivalis* infection, which was partially mitigated by *Zbp1* siRNAs (Fig. 4d).

Previous studies have suggested the role of the ZBP1–receptor-interacting protein kinase 3 (RIPK3) complex in triggering pyroptosis and apoptosis through the recruitment and activation of cysteine–aspartic acid protease 8 (Caspase8)^15,27^. In addition, RIPK3 is associated with the phosphorylation and subsequent activation of mixed-lineage kinase domain-like protein (MLKL), leading to necrosis^28^. In this study, the siRNA targeting *Zbp1* significantly reduced the *P. gingivalis*-induced upregulation of Zbp1 expression (Supplementary Fig. 4a). Importantly, *Zbp1* knockdown resulted in a reduction in the levels of p-Ripk3, cleaved Caspase8, cleaved cysteine–aspartic acid protease 3 (Caspase3, a crucial enzyme in the apoptosis pathway), NOD-like receptor pyrin domain-containing 3 (Nlrp3) and cleaved Gasdermin D (Gsdmd, a key protein in the pyroptosis pathway), and p-Mlkl (a crucial protein in the necroptosis pathway) in *P. gingivalis*-infected BMDMs (Fig. 4e, f), indicating a comprehensive inhibition of PANoptosis pathways. Consistently, IF assays showed decreased expression of cleaved Gsdmd and cleaved Caspase3 in the *Zbp1* siRNA groups infected with *P. gingivalis* (Supplementary Fig. 4b). Furthermore, the detection of p-Mlkl with the antibody revealed distinct localization of p-Mlkl fluorescence on the plasma membrane upon *P. gingivalis* stimulation, consistent with the translocation observed during necrotic activation. By contrast, co-transfection with *Zbp1* siRNAs reduced the plasma membrane localization of p-Mlkl (Supplementary Fig. 4c). Together, these results suggested that *Zbp1* knockdown inhibited the activation of *P. gingivalis*-mediated macrophage pyroptosis, apoptosis and necrosis, further confirming the crucial role of *Zbp1* in *P. gingivalis*-induced PANoptosis.

During bacterial infections, host immune cells are activated, leading to elevated oxidative stress levels. Excessive reactive oxygen species (ROS) can compromise mitochondrial function by increasing the permeability of the mitochondrial outer membrane, facilitating the release of mtDNA into the cytoplasm. Given the DNA recognition properties of ZBP1, the released mtDNA may be recognized by ZBP1, triggering the activation of downstream signaling pathways. Therefore, investigating the changes in mitochondrial function is essential for elucidating the mechanism of ZBP1-mediated cell death signal amplification during bacterial infections^29^. Figure 4g demonstrated a significant increase of ROS levels in BMDMs after *P. gingivalis* infection. 5,5′,6,6′-Tetrachloro-1,1′,3,3′-tetraethyl-imidacarbocyanine (JC-1) staining indicated a decreased red/green fluorescence ratio after *P. gingivalis* infection, suggesting a notable decline in mitochondrial membrane potential (Δ*ψ*_M_) (Fig. 4h). In addition, transmission electron microscopy revealed swelling mitochondrial morphology in infected BMDMs (Fig. 4i). MitoSOX staining and adenosine triphosphate (ATP) detection suggested elevated mitochondrial ROS (mtROS) levels and impaired ATP production in the infected BMDMs (Fig. 4j, k). IF staining of MitoTracker showed increased fragmentation of mitochondrial morphology upon *P. gingivalis* stimulation (Fig. 4l). PCR assays revealed statistically significant cytoplasmic expression of mitochondrial genes *Cyt B* and *Nd2* after *P. gingivalis* infection, confirming the release of mtDNA into the cytoplasm (Fig. 4m).

To determine whether mtDNA can bind to the Zα domains of Zbp1 and facilitate downstream signal amplification, a ChIP experiment using Zbp1 antibody was conducted. As depicted in Fig. 4n, the detection of *Cyt B* and *Nd2* in the Zbp1 immunoprecipitates of the *P. gingivalis*-stimulated group indicated interactions between mtDNA and Zbp1 (Fig. 4n). Furthermore, depleting mtDNA with ethidium bromide (EtBr) led to undetectable levels of *Cyt B* and *Nd2* in Zbp1-bound immunoprecipitates (Fig. 4o) and reduced expression of PANoptosis-related indicators (Supplementary Fig. 4d,e). In summary, *P. gingivalis* infection induced mitochondrial oxidative stress and promoted mtDNA release, thereby amplifying *Zbp1*-mediated downstream PANoptosis signaling.

### Deletion of Zbp1 protected against PANoptosis activation and periodontal tissue destruction in *P. gingivalis*-induced mouse periodontitis

**Fig. 5:**
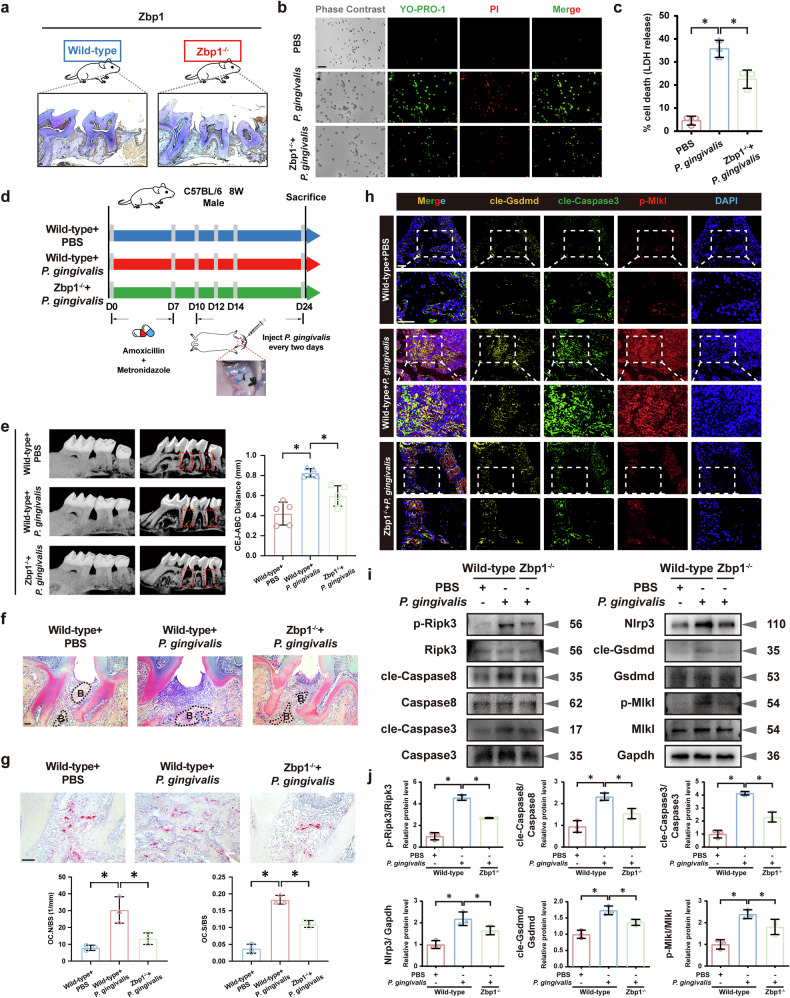
Deletion of Zbp1 protected against PANoptosis activation and periodontal tissue destruction in *P. gingivalis*-induced mouse periodontitis. **a** IHC staining confirming Zbp1 deficiency in periodontal tissues of *Zbp1*^−/−^ mice. Scale bar, 50 μm. Decreased apoptosis, necrosis (**b**) and LDH release (**c**) in BMDMs from *Zbp1*^−/−^ mice. *n* = 3. Scale bar, 50 μm. **d** Establishment of a mouse periodontitis model in WT and *Zbp1*^−/−^ mice. *n* = 5. **e** Representative micro-CT images revealing diminished periodontal tissue destruction in *Zbp1*^−/−^ mice. *n* = 5. Diminished periodontal tissue destruction (**f**) and reduced osteoclastogenesis (**g**) in *Zbp1*^−/−^ mice after *P. gingivalis* challenge. *n* = 5. Scale bar, 50 μm. **h** Reduced cleaved Gsdmd, cleaved Caspase3 and p-Mlkl expression in periodontitis lesions of *Zbp1*^−/−^ mice. Scale bar, 50 μm. **i**, **j** Protein analysis from periodontal tissues showing Zbp1 deficiency alleviating upregulation of p-Ripk3, cleaved Caspase8, cleaved Caspase3, Nlrp3, cleaved Gsdmd and p-Mlkl induced by *P. gingivalis* infection. *n* = 3. All data were derived from independent experiments. *P < 0.05.

To further elucidate the regulatory role of *Zbp1*-mediated PANoptosis in *P. gingivalis*-induced periodontitis, *Zbp1*-knockout mice (m*Zbp1*KO, *Zbp1*^−/−^) mice were generated and IHC staining confirmed the deficiency of Zbp1 in the periodontal tissues (Fig. 5a). The levels of IL-1β and TNF-α after *P. gingivalis* infection decreased in the BMDMs derived from *Zbp1*^−/−^ mice compared with those from WT mice (Supplementary Fig. 5a, b). The YO-PRO-1/PI staining assay further verified that BMDMs derived from *Zbp1*^−/−^ mice showed marked inhibition of macrophage apoptosis and death induced by *P. gingivalis* infection (Fig. 5b), and a similar expression pattern of LDH was observed (Fig. 5c).

Based on a ligature-induced mouse model of periodontitis, *P. gingivalis* was injected vertically into the alveolar crest of WT and *Zbp1*^−/−^ mice (Fig. 5d). *Zbp1*^−/−^ mice exhibited diminished periodontal destruction, reduced osteoclastogenesis and reduced proinflammatory cytokine expression (Fig. 5e–g and Supplementary Fig. 5c,d). IF staining showed reduced expression of cleaved Gsdmd, cleaved Caspase3 and p-Mlkl in periodontitis tissues from *Zbp1*^−/−^ mice (Fig. 5h). Furthermore, protein analysis from periodontal tissues revealed that Zbp1 deficiency mitigated the upregulation of PANoptosis signaling stimulated by *P. gingivalis* (Fig. 5i, j). These findings suggest that the specific deletion of Zbp1 protects against PANoptosis activation and periodontal tissue destruction in *P. gingivalis-*induced periodontitis.

### *P. gingivalis* activated Zbp1 in BMDMs via the Tlr2/4–JNK pathways

**Fig. 6:**
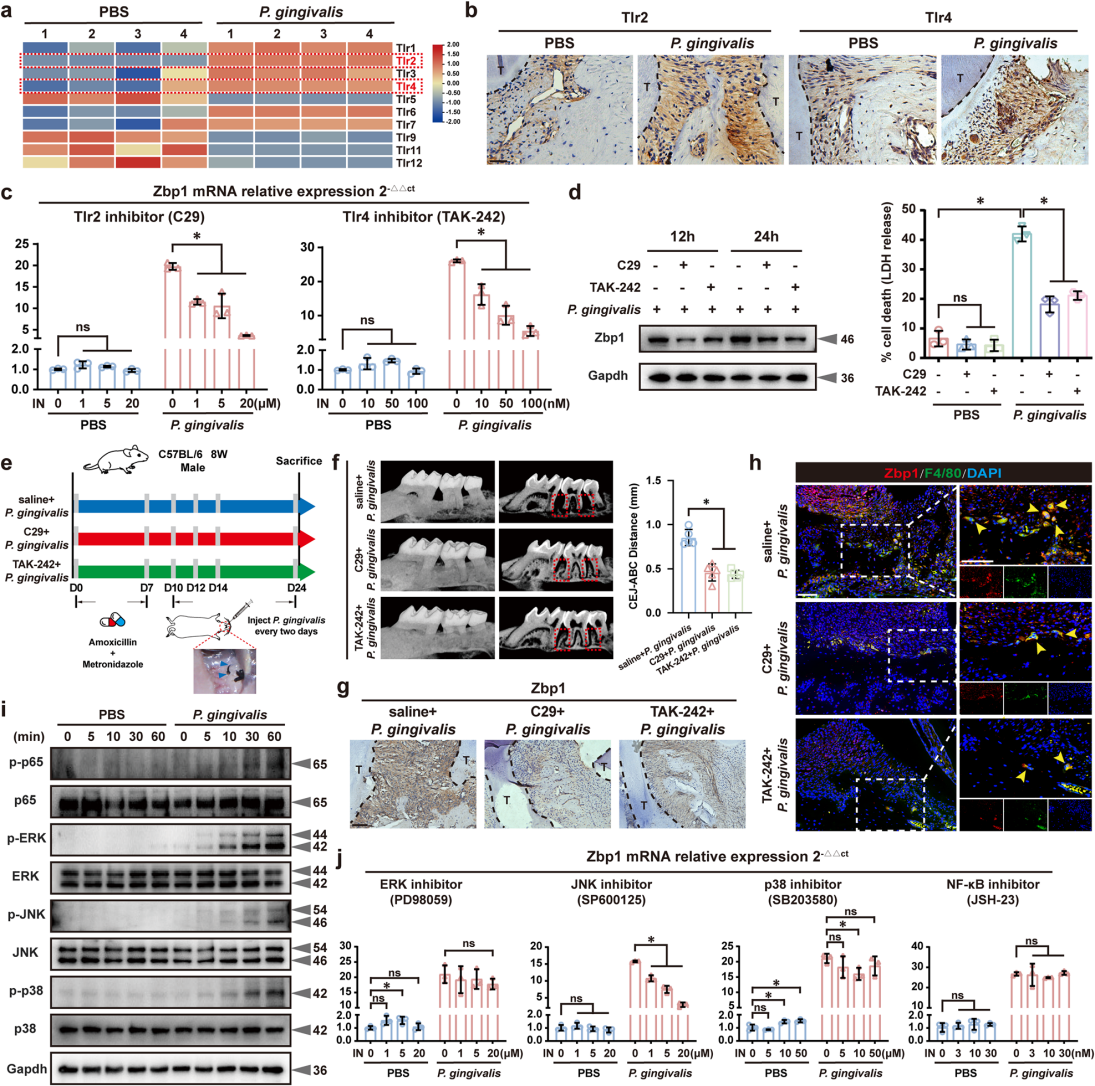
*P. gingivalis* activated Zbp1 in BMDMs via the Tlr2/4–JNK pathways. **a** A heat map showing Tlr gene expression profiles in BMDMs stimulated with *P. gingivalis*. **b** IHC assays revealing increased levels of Tlr2 and Tlr4 in *P. gingivalis*-induced periodontal tissues. *n* = 5. Scale bar, 50 μm. Tlr2 and Tlr4 inhibitors effectively counteracted Zbp1 gene (**c**) and protein expression (**d**) in the infected BMDMs*. n* = 3. **e** *P. gingivalis*-infected mouse periodontitis model with daily intraperitoneal administration of C29 (5 mg/kg) and TAK-242 (3 mg/kg)*. n* = 5. Micro-CT (**f**), IHC (**g**) and IF staining (**h**) showing reduced alveolar bone resorption and Zbp1 activation with C29 and TAK-242 treatment. *n* = 5. Scale bar, 50 μm. **i** Western blotting showing increased phosphorylation levels of p65, ERK, JNK and p38 in BMDMs after *P. gingivalis* stimulation*. n* = 3. **j** The JNK inhibitor dose-dependently inhibited Zbp1 expression in the infected BMDMs. *n* = 3. All data were derived from independent experiments. ns, no significance, *P < 0.05.

Previous studies have highlighted the pivotal role of Toll-like receptor (TLR) family in mediating interactions between microbial flora and immune cells^30^. Our KEGG enrichment analysis verified substantial activation of the TLR pathway in *P. gingivalis*-stimulated BMDMs (Fig. 3e). To further investigate the pattern recognition receptors responsible for macrophage recognition by *P. gingivalis*, we filtered the relative expression of Tlr genes (Fig. 6a) and identified pronounced upregulation of *Tlr2* and *Tlr4* by qRT–PCR (Supplementary Fig. 6a). Myeloid differentiation primary response 88 (Myd88) is an essential bridging protein for downstream signaling by all TLRs except Tlr3 (ref. ^31^). In this study, IHC assays further demonstrated that *P. gingivalis* increased Tlr2, Tlr4 and Myd88 expression in *P. gingivalis-*induced mouse periodontitis compared with that in the controls (Fig. 6b and Supplementary Fig. 6b). Furthermore, specific inhibitors targeting the Tlr2 (C29), Tlr4 (TAK-242) and Myd88 (T6167923) pathways effectively counteracted the induction of *Zbp1* mRNA and protein expression in BMDMs stimulated with *P. gingivalis* (Fig. 6c, d and Supplementary Fig. 6c). YO-PRO-1/PI staining confirmed that treatment with C29 and TAK-242 significantly suppressed apoptosis and necrosis induction in *P. gingivalis*-stimulated BMDMs (Supplementary Fig. 6d). Similar observations were made by IF staining of cleaved Gsdmd, cleaved Caspase3 and p-MLKL (Supplementary Fig. 6e,f). Importantly, daily systemic administration via intraperitoneal injection of C29 (5 mg/kg) and TAK-242 (3 mg/kg) protected against increased alveolar bone resorption and Zbp1 activation in mice infected with *P. gingivalis* (Fig. 6e–h).

Tlr–Myd88 pathway activation can signal downstream to NF-κB (p65) and MAPK (including ERK, JNK and p38) pathways, leading to the production of proinflammatory cytokines^32^. In this study, KEGG enrichment analysis indicated that p65 and MAPK pathways were enriched after *P. gingivalis* infection (Fig. 3e). Western blotting assay further demonstrated that *P. gingivalis* stimulation increased the phosphorylation of p65, ERK, JNK and p38 in BMDMs (Fig. 6i). Notably, noncytotoxic doses of a JNK inhibitor (SP600125) significantly inhibited Zbp1 expression in *P. gingivalis*-induced BMDMs in a dose-dependent manner, indicating a predominant role for the JNK signaling pathway downstream of Tlr2/4 in controlling *Zbp1* transcription (Fig. 6j). These findings demonstrate that Tlr2/4 is involved in the recognition of *P. gingivalis* by macrophages, which subsequently activates the JNK pathway, leading to the activation of *Zbp1* and driving periodontal inflammation and bone resorption.

### *P. gingivalis* activated transcription factors (TFs) Stat3 and Stat5 to promote *Zbp1* transcription

**Fig. 7:**
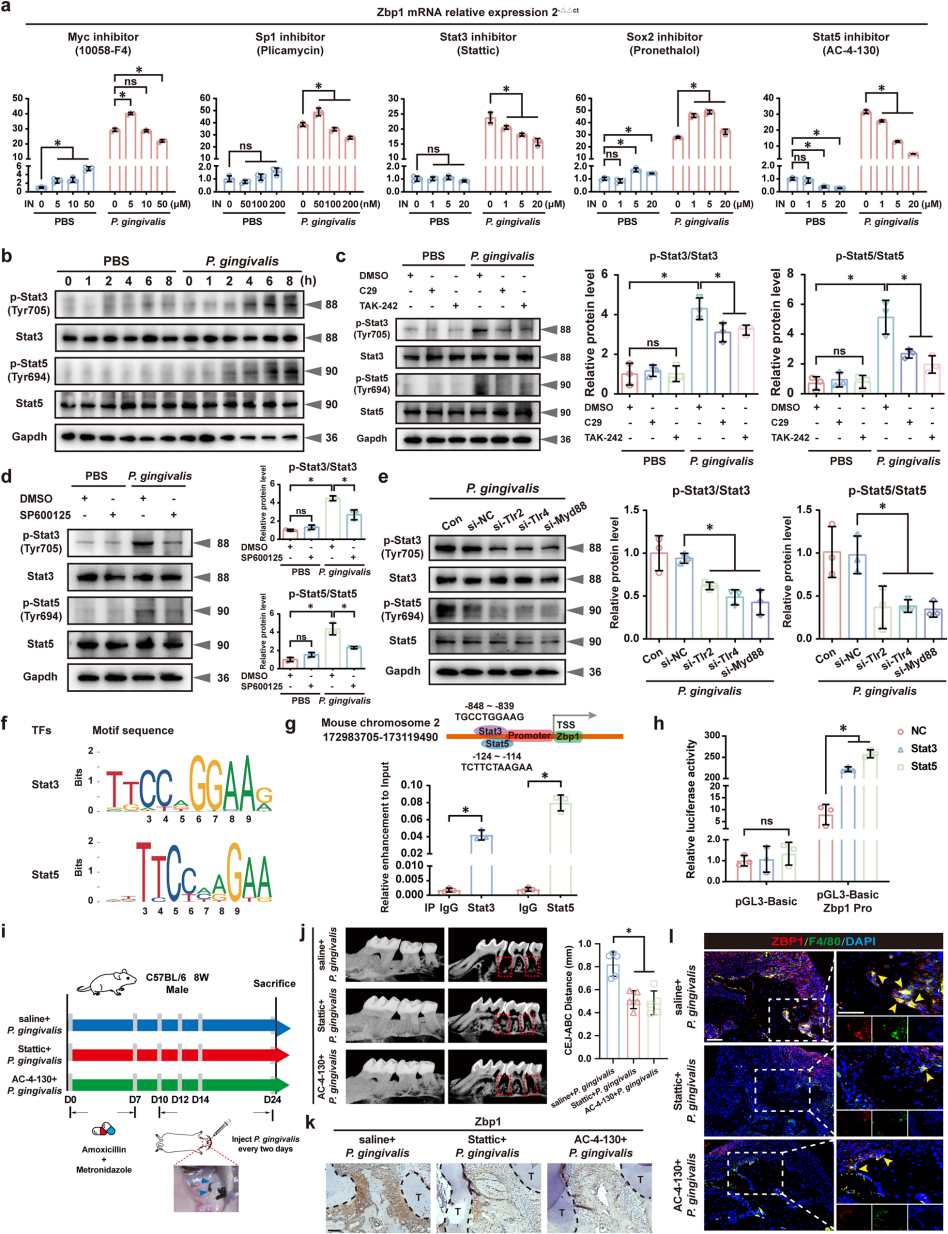
*P. gingivalis* activated TFs Stat3 and Stat5 to promote *Zbp1* transcription. **a** *P. gingivalis*-induced BMDMs showing decreased Zbp1 expression with Stattic and AC-4-130 treatment. *n* = 3. **b** Western blotting showing increased phosphorylation of Stat3 and Stat5 after *P. gingivalis* infection. *n* = 3. Pharmacological inhibition (**c**, **d**) and genetic depletion (**e**) of the Tlr2/Tlr4–JNK pathway reduced the phosphorylation of Stat3 and Stat5 in *P. gingivalis*-stimulated BMDMs. *n* = 3. **f** Putative binding sites of Stat3 and Stat5 in the Zbp1 promoter regions. **g** ChIP assays revealing significant Stat3 and Stat5 enrichment in the predicted binding regions of Zbp1 promoter. *n* = 3. **h** Luciferase reporter assays validating the regulatory roles of Stat3 and Stat5 in Zbp1 transcription. *n* = 3. **i** *P. gingivalis*-infected mouse periodontitis model with daily intraperitoneal administration of Stattic (5 mg/kg) and AC-4-130 (5 mg/kg)*. n* = 5. Micro-CT images (**j**), IHC (**k**) and IF staining (**l**) showing decreased alveolar bone resorption and Zbp1 activation with Stattic and AC-4-130 treatment. *n* = 5. Scale bar, 50 μm. All data were derived from independent experiments. ns, no significance, *P < 0.05.

To delve into the intricate mechanism by which Tlr2/4–JNK promotes Zbp1 activation after *P. gingivalis* stimulation, we conducted a detailed bioinformatics analysis to identify potential TFs involved in Zbp1 transcription using the Jasper database (https://jaspar.genereg.net/). The predicted scores indicated that *Zbp1* transcription may be governed by the binding of multiple TFs, including Myc, SP1, Stat3, Sox2 and Stat5 (Supplementary Fig. 7a). We subsequently performed qRT–PCR and found that the increased *Zbp1* expression was blunted by Stat3 inhibitor (Stattic) and Stat5 inhibitor (AC-4-130), implying that Stat3 and Stat5 are crucial TFs for *Zbp1* induction (Fig. 7a). Western blotting assays demonstrated that *P. gingivalis* infection significantly enhanced the phosphorylation levels of Stat3 and Stat5 (Fig. 7b). In addition, IF staining revealed the nuclear translocation of Stat3 and Stat5 in BMDMs after *P. gingivalis* infection (Supplementary Fig. 7b,c). More importantly, pharmacological blocking and genetic depletion of the Tlr2/Tlr4–JNK pathway were shown to block the phosphorylation of Stat3 and Stat5, confirming their activation by the Tlr2/Tlr4–JNK pathway in *P. gingivalis*-stimulated BMDMs (Fig. 7c–e).

Further analysis of the putative binding sites of Stat3 and Stat5 in the Zbp1 promoter revealed predicted binding regions with high affinity score (Fig. 7f). Primers were designed accordingly, and a ChIP assay was performed using specific Stat3 and Stat5 antibodies. The qRT–PCR showed statistically significant difference in enrichment of Zbp1 in this binding region compared with the IgG control (Fig. 7g). Furthermore, luciferase reporters were constructed to insert the Zbp1 promoter DNA sequence into the reporter gene vector (pGL3-Basic). Results showed that co-transfection with Stat3 and Stat5 plasmids enhanced luciferase activity, validating their regulatory roles in Zbp1 transcription (Fig. 7h). In a mouse model of periodontitis, daily intraperitoneal administration of Stat3 (5 mg/kg) and Stat5 (5 mg/kg) inhibitors significantly reduced *P. gingivalis*-induced periodontal resorption and Zbp1 induction (Fig. 7i-l). Collectively, these findings suggested that Stat3 and Stat5 activation mediated by the Tlr2/Tlr4–JNK pathway regulated Zbp1 induction in *P. gingivalis*-stimulated BMDMs.

### MNs delivery of Sal B alleviated *P. gingivalis*-induced periodontal resorption and promoted mucosal wound healing

**Fig. 8:**
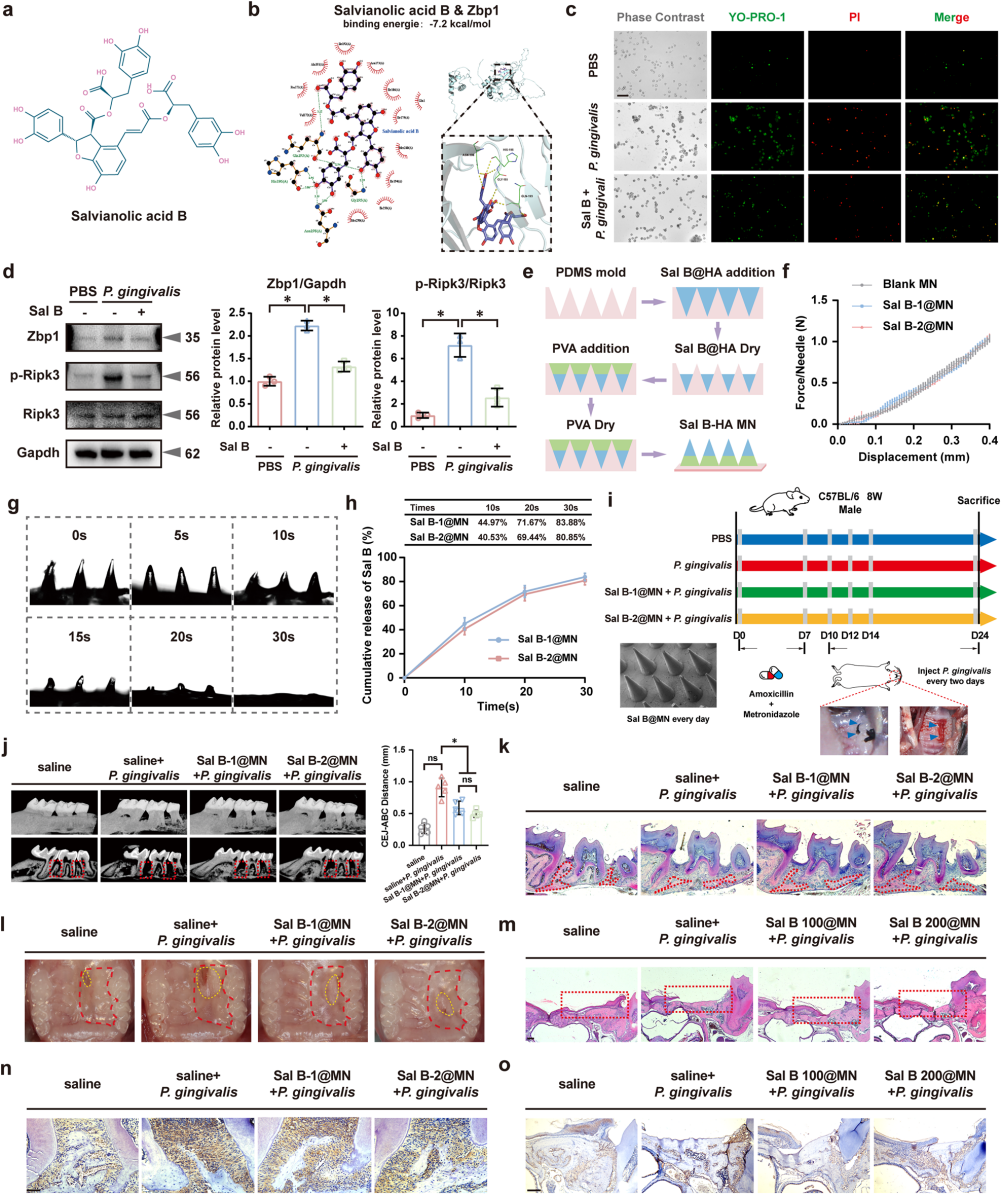
MNs delivery of Sal B alleviated *P. gingivalis*-induced periodontal resorption and promoted mucosal wound healing. **a** The chemical structure of Sal B. **b** Molecular docking simulations revealing Sal B binds favorably to Zbp1. **c** YO-PRO-1/PI staining showing inhibited apoptosis and necrosis with Sal B intervention. *n* = 3. Scale bar, 50 μm. **d** Sal B treatment significantly reduced Zbp1 and p-Ripk3 levels. *n* = 3. **e** Encapsulation of Sal B (0.5 mg or 1 mg) into PVA MN patches. **f** Sal B@MNs demonstrating mechanical strength exceeding 1 N per needle. **g** Dissolution of the modified MN tips in PBS. **h** Delivery efficiency of Sal B-1@MN and Sal B-2@MN. *n* = 3. **i** *P. gingivalis*-infected mouse periodontitis and hard palate wound healing models with daily administration of Sal B@MNs. *n* = 5. Representative images and HE staining illustrating reduced periodontal tissue resorption (**j**, **k**) and and near-complete wound healing (**l**, **m**) in mice treated with Sal B@MNs. *n* = 5. Scale bar, 50 μm. IHC images showing lower Zbp1 expression in the periodontitis (**n**) and hard palate wound healing (**o**) tissues of Sal B@MNs-treated mice. *n* = 5. Scale bar, 50 μm. All data were derived from independent experiments. *P < 0.05.

To expand our search for potential bioactive compounds targeting Zbp1, we utilized the Comparative Toxicogenomics Database (https://pwaqa40.ctdbase.org/) and predicted the interactions between Danshen extract and the target protein Zbp1 (Supplementary Fig. 8a). Danshen is a traditional Chinese medicine widely used to prevent and treat vascular diseases^33^. Subsequently, a water-soluble polyphenolic antioxidant extracted from Danshen—Sal B (Fig. 8a)—was chosen for subsequent investigation owing to its reported inhibition of proinflammatory pathways^34,35^. Molecular docking simulations were performed to explore the interaction between Sal B and Zbp1 (Fig. 8b), revealing binding energies lower than −5 kcal/mol (−7.2 kcal/mol), indicative of a favorable binding activity. In addition, in vitro experiments showed that 50 μM Sal B was nontoxic and notably suppressed the upregulation of inflammatory markers induced by *P. gingivalis* (Supplementary Fig. 8b,c). Further characterization of YO-PRO-1/PI staining revealed that Sal B intervention inhibited apoptosis and death associated with *P. gingivalis* stimulation (Fig. 8c). Western blotting assays indicated that Sal B treatment significantly reduced Zbp1 and p-Ripk3 levels (Fig. 8d), leading to inhibited downstream PANoptosis signal transduction (Supplementary Fig. 8d,e).

MNs have been reported as a novel physical transdermal technology, effectively facilitating drug delivery^36^. In this study, we encapsulated either 0.5 mg or 1 mg Sal B into a 10 × 10 array of the PVA MN patches (labeled as Sal B-1@MN and Sal B-2@MN), respectively (Fig. 8e). The MNs exhibited a mechanical strength exceeding 1 N per needle, indicating their ability to penetrate the oral mucosal tissue (Fig. 8f). In vitro experiments confirmed the dissolution of the modified MN tips in PBS within 30 s, whereas the backing layer remained intact (Fig. 8g). The delivery efficiency was further validated before and after MN immersion in PBS for 30 s, reaching up to 83.88% and 80.85% for Sal B-1@MN and Sal B-2@MN, respectively (Fig. 8h). We subsequently established a ligature-induced periodontitis model and a hard palate wound healing model in mice, in which a *P. gingivalis* suspension was injected vertically onto the alveolar crest or wound edge every other day (Fig. 8i). Sal B@MNs were administered daily to the local mucosa surrounding periodontitis or palatal wounds. By day 14 after model establishment, the mice treated with *P. gingivalis* and Sal B @MN showed reduced periodontal tissue resorption (Fig. 8j, k) and nearly complete wound healing (Fig. 8l, m). No notable difference was observed in the therapeutic effects of the two concentrations of Sal B. IHC images revealed a lower expression of Zbp1 in the periodontal and palatal mucosa tissues of Sal B @MN treatment mice than in the *P. gingivalis*-stimulated groups (Fig. 8n, o). In summary, these findings suggest that the local delivery of Sal B by MNs might prevent *P. gingivalis*-mediated *Zbp1* activation, thereby suppressing periodontal inflammation and tissue resorption.

## Discussion

Macrophages are crucial for combating persistent infections and providing protection to infected tissues. However, pathogen-induced cell death during this process can lead to damage and severe disease^37^. Accumulating evidence indicates that different death processes are not mutually exclusive; instead, they interact as a collective series of events^7^. Although apoptosis has traditionally been considered immunologically silent, studies on the crosstalk between apoptosis, pyroptosis and necroptosis suggest that apoptotic cell death can also cause inflammation in certain cell types^38^. In this study, we propose a novel pathogenic model of *P. gingivalis*-induced periodontitis, suggesting that *P. gingivalis* activates Zbp1-mediated PANoptosis in macrophages, thereby contributing to periodontal tissues damage. More importantly, Zbp1 deletion in mice alleviated periodontal cell death and tissue resorption. Therefore, Zbp1 may be a crucial therapeutic target for blocking proinflammatory cell death and preventing *P. gingivalis*-mediated periodontal damage.

Under conditions of periodontitis, sustained inflammation and tissue damage contribute to elevated oxidative stress^39^. The interaction of *P. gingivalis* lipopolysaccharides and ATP has been shown to elevate intracellular ROS levels, activating NF-κB–NLRP3 signaling and inducing gingival fibroblast death^40^. In this study, we validated the involvement of mtROS disruption and mtDNA, into the cytoplasm. Cytoplasmic mtDNA acts as an intracellular damage signal recognized by DNA sensors, triggering inflammatory pathways^41^. Our investigation revealed the interaction of Zbp1 with mtDNA, eliciting downstream PANoptosis signal amplification. In addition, studies have reported that intracellular receptor cGAS can bind to sting, further catalyzing cGAMP production, thereby activating the STING pathway and ultimately facilitating intracellular inflammatory responses and programmed immune cell death^42^. Furthermore, cytoplasmic mtDNA release can directly activate the NLRP3 complex, prompting the maturation and release of proinflammatory cytokine IL-1β and IL-18, further propagating cellular apoptosis^43^. Therefore, a future research direction may involve a more comprehensive elucidation of the complex mechanisms controlling oxidative stress and cell death in periodontal pathology.

Tlr2 and Tlr4 are well established as central pathogens recognition receptors in immune cells^30^. Bacterial pathogen-associated molecular patterns activate the Tlr2/Tlr4–Myd88 pathway in pathological environments, converting infection signals into bone resorption signals, activating RANKL expression and inducing bone destruction^44^. However, the precise role of these receptors in periodontitis-related cell death remains unclear. Our data suggested that the activation of Tlr2/4 was essential for the recognition of *P. gingivalis* by macrophages, mediating Zbp1-activated downstream cell death pathways. Mechanistically, activation of the Tlr2/Tlr4–JNK pathway increased the phosphorylation of Stat3 and Stat5, and blocking these TFs prevented *Zbp1* induction in macrophages. Studies have reported that Stat3 and Stat5 facilitate the transcription of various inflammatory mediators, including IL-6 and IL-17, by directly targeting their promoter regions, thereby amplifying the inflammatory response^45^. However, their involvement in cell-death-related pathways is multifaceted. Under normal conditions, Stat3 and Stat5 serve as antiapoptotic factors, shielding cells from damage^46^. Conversely, in certain circumstances, the activation of Stat3 and Stat5 may also contribute to the modulation of inflammation-associated cell death processes, promoting apoptosis and necrosis^47^. This duality could be influenced by factors such as cell type, environmental cues and upstream signaling pathway regulation, warranting further investigation in specific contexts.

Sal B is a bioactive component isolated from Danshen, involving diverse anti-inflammatory mechanisms^33^. Functioning as a natural antioxidant, Sal B demonstrates substantial antioxidative activity, mitigating the adverse effects of oxidative stress during inflammation^48^. Furthermore, Sal B can modulate various inflammation-related signals, including NF-κB, MAPK and STAT pathways^35,49^. Studies have also indicated that Sal B administration reduces the prevalence of M1-polarized macrophages and alleviates the release of inflammatory factors^50^. In this study, MNs loaded with Sal B have been developed to facilitate drug penetration. Upon penetration of the tissues by the crosslinked MNs, the interstitial fluid swelled, leading to the release of Sal B loaded inside through free diffusion. The treatment inhibited local Zbp1 expression, reduced periodontal tissue damage and enhanced healing of the palatal mucosa. Studies have demonstrated that Sal B can influence protein expression through several pathways, including: (1) promoting protein degradation, such as via the ubiquitin-proteasome pathway^51^; (2) inhibiting transcription or translation, thus reducing protein synthesis^52^; and (3) altering protein stability or intracellular localization^53^. Based on the finding that Sal B reduces Zbp1 protein expression, we hypothesize that Sal B may directly bind to Zbp1, inducing conformational changes that lead to its degradation or disrupt its interactions with other molecules; however, further investigations are required to elucidate the precise mechanism. Overall, our findings suggest that Sal B may offer novel therapeutic potential for periodontitis and other inflammatory diseases of the oral cavity, expanding its current applications.

Collectively, our results highlight the crucial role of Zbp1-mediated PANoptosis in periodontitis. As a crucial oral pathogen, *P. gingivalis* triggers a mitochondrial stress response, leading to the release of mtDNA. This mtDNA then interacte with Zbp1, consequently augmenting its downstream PANoptosis signals. In addition, *P. gingivalis* stimulates macrophage Zbp1 expression through the Tlr2/4–JNK–Stat3/5 pathway, exacerbating periodontal inflammation and tissue destruction. The pharmacological delivery of Sal B through MNs effectively inhibited Zbp1 levels, prevented severe periodontal damage. In conclusion, Sal B@MNs and other drugs targeting Zbp1 and the downstream regulatory mechanism of PANoptosis may represent new strategies for alleviating periodontal tissue destruction and treating periodontitis. These approaches hold promise for advancing therapeutic interventions for periodontal health.

## Supplementary information

Supplementary Information

## Data Availability

The single-cell sequencing dataset GSE171213 is publicly available in the GEO database (https://www.ncbi.nlm.nih.gov/geo/). All data are available from the corresponding author on reasonable request.

## References

[1] Slots, J. Periodontitis: facts, fallacies and the future. *Periodontol. 2000* **75**, 7–23 (2017). 28758294 10.1111/prd.12221

[2] Huang, X., Li, Y., Zhang, J. & Feng, Q. Linking periodontitis with inflammatory bowel disease through the oral–gut axis: the potential role of *Porphyromonas gingivalis*. *Biomedicines* **12**, 685 (2024). 38540299 10.3390/biomedicines12030685 PMC10968003

[3] Lunar Silva, I. & Cascales, E. Molecular strategies underlying *Porphyromonas gingivalis* virulence. *J. Mol. Biol.* **433**, 166836 (2021). 33539891 10.1016/j.jmb.2021.166836

[4] Arafa, E. I. et al. Recruitment and training of alveolar macrophages after pneumococcal pneumonia. *JCI Insight* **7**, e150239 (2022). 35133985 10.1172/jci.insight.150239 PMC8983128

[5] Galli, G. & Saleh, M. Immunometabolism of macrophages in bacterial infections. *Front. Cell Infect. Microbiol.* **10**, 607650 (2020). 33585278 10.3389/fcimb.2020.607650 PMC7879570

[6] Groeger, S. & Meyle, J. The role of programmed death receptor (PD-)1/PD-ligand (L)1 in periodontitis and cancer. *Periodontol. 2000*10.1111/prd.12548 (2024). 10.1111/prd.12548 PMC11579837 38351432

[7] Frank, D. & Vince, J. E. Pyroptosis versus necroptosis: similarities, differences, and crosstalk. *Cell Death Differ.* **26**, 99–114 (2019). 30341423 10.1038/s41418-018-0212-6 PMC6294779

[8] He, Y. et al. Glycolytic reprogramming controls periodontitis-associated macrophage pyroptosis via AMPK/SIRT1/NF-κB signaling pathway. *Int. Immunopharmacol.* **119**, 110192 (2023). 37068341 10.1016/j.intimp.2023.110192

[9] Luo, W. et al. The role of macrophage death in periodontitis: a review. *Inflammation*10.1007/s10753-024-02015-4 (2024). 10.1007/s10753-024-02015-4 38691250

[10] Yang, Y., Wang, L., Zhang, H. & Luo, L. Mixed lineage kinase domain-like pseudokinase-mediated necroptosis aggravates periodontitis progression. *J. Mol. Med.* **100**, 77–86 (2022). 34647144 10.1007/s00109-021-02126-7

[11] Malireddi, R. K. S., Kesavardhana, S. & Kanneganti, T.-D. ZBP1 and TAK1: master regulators of NLRP3 inflammasome/pyroptosis, apoptosis, and necroptosis (PAN-optosis). *Front. Cell Infect. Microbiol.* **9**, 406 (2019). 31850239 10.3389/fcimb.2019.00406 PMC6902032

[12] Jiang, W., Deng, Z., Dai, X. & Zhao, W. PANoptosis: a new insight into oral infectious diseases. *Front. Immunol.* **12**, 789610 (2021). 34970269 10.3389/fimmu.2021.789610 PMC8712492

[13] Zhao, X. et al. ZBP1 (DAI/DLM-1) promotes osteogenic differentiation while inhibiting adipogenic differentiation in mesenchymal stem cells through a positive feedback loop of Wnt/β-catenin signaling. *Bone Res.* **8**, 12 (2020). 32195010 10.1038/s41413-020-0085-4 PMC7058036

[14] Malireddi, R. K. S., Sharma, B. R., Bynigeri, R. R., Wang, Y., Lu, J. & Kanneganti, T.-D. ZBP1 drives IAV-induced NLRP3 inflammasome activation and lytic cell death, PANoptosis, independent of the necroptosis executioner MLKL. *Viruses* **15**, 2141 (2023). 38005819 10.3390/v15112141 PMC10674287

[15] Jiao, H. et al. Z-nucleic-acid sensing triggers ZBP1-dependent necroptosis and inflammation. *Nature* **580**, 391–395 (2020). 32296175 10.1038/s41586-020-2129-8 PMC7279955

[16] Wang, Z., Chen, H., Peng, L., He, Y. & Zhang, X. Revealing a potential necroptosis-related axis (RP11-138A9.1/hsa-miR-98-5p/ZBP1) in periodontitis by construction of the ceRNA network. *J. Periodontal Res.* **58**, 968–985 (2023). 37357608 10.1111/jre.13157

[17] Liu, H., Liu, Y.-X., Fan, W. & Fan, B. Metformin switches cell death modes to soothe the apical periodontitis via ZBP1. *FASEB J.* **38**, e23549 (2024). 38446465 10.1096/fj.202302073R

[18] Tonetti, M. S., Greenwell, H. & Kornman, K. S. Staging and grading of periodontitis: framework and proposal of a new classification and case definition. *J. Periodontol.* **89**, S159–S172 (2018). 29926952 10.1002/JPER.18-0006

[19] Marchesan, J. et al. An experimental murine model to study periodontitis. *Nat. Protoc.* **13**, 2247–2267 (2018). 30218100 10.1038/s41596-018-0035-4 PMC6773250

[20] Mugri, M. H. Efficacy of systemic amoxicillin–metronidazole in periodontitis patients with diabetes mellitus: a systematic review of randomized clinical trials. *Medicina* **58**, 1605 (2022). 36363562 10.3390/medicina58111605 PMC9695465

[21] Zou, Y. et al. SIRT6 inhibition delays peripheral nerve recovery by suppressing migration, phagocytosis and M2-polarization of macrophages. *Cell Biosci.* **11**, 210 (2021). 34906231 10.1186/s13578-021-00725-y PMC8672560

[22] Evavold, C. L. et al. Control of gasdermin D oligomerization and pyroptosis by the Ragulator–Rag–mTORC1 pathway. *Cell* **184**, 4495–4511 (2021). 34289345 10.1016/j.cell.2021.06.028 PMC8380731

[23] Chen, Y. et al. Single-cell RNA landscape of the osteoimmunology microenvironment in periodontitis. *Theranostics* **12**, 1074–1096 (2022). 35154475 10.7150/thno.65694 PMC8771561

[24] Zhang, W. et al. Chimeric antigen receptor macrophage therapy for breast tumours mediated by targeting the tumour extracellular matrix. *Br. J. Cancer* **121**, 837–845 (2019). 31570753 10.1038/s41416-019-0578-3 PMC6889154

[25] Alam, M. I. et al. NLRP3 inflammasome negatively regulates RANKL-induced osteoclastogenesis of mouse bone marrow macrophages but positively regulates it in the presence of lipopolysaccharides. *Int J. Mol. Sci.* **23**, 6096 (2022). 35682777 10.3390/ijms23116096 PMC9181162

[26] Karki, R. et al. ZBP1-dependent inflammatory cell death, PANoptosis, and cytokine storm disrupt IFN therapeutic efficacy during coronavirus infection. *Sci. Immunol.* **7**, eabo6294 (2022). 35587515 10.1126/sciimmunol.abo6294 PMC9161373

[27] Yang, D. et al. ZBP1 mediates interferon-induced necroptosis. *Cell Mol. Immunol.* **17**, 356–368 (2020). 31076724 10.1038/s41423-019-0237-x PMC7109092

[28] Zhang, X. et al. RIPK3-MLKL necroptotic signalling amplifies STING pathway and exacerbates lethal sepsis. *Clin. Transl. Med* **13**, e1334 (2023). 37475188 10.1002/ctm2.1334 PMC10359592

[29] Lei, Y. et al. Cooperative sensing of mitochondrial DNA by ZBP1 and cGAS promotes cardiotoxicity. *Cell* **186**, 3013–3032 (2023). 37352855 10.1016/j.cell.2023.05.039 PMC10330843

[30] Duan, T., Du, Y., Xing, C., Wang, H. Y. & Wang, R.-F. Toll-like receptor signaling and its role in cell-mediated immunity. *Front. Immunol.* **13**, 812774 (2022). 35309296 10.3389/fimmu.2022.812774 PMC8927970

[31] Saleki, K., Alijanizadeh, P., Javanmehr, N. & Rezaei, N. The role of Toll-like receptors in neuropsychiatric disorders: Immunopathology, treatment, and management. *Med. Res. Rev.*10.1002/med.22012 (2024). 38226452 10.1002/med.22012

[32] Dawuti, A. et al. Salvianolic acid A alleviates heart failure with preserved ejection fraction via regulating TLR/Myd88/TRAF/NF-κB and p38MAPK/CREB signaling pathways. *Biomed. Pharmacother.* **168**, 115837 (2023). 37931518 10.1016/j.biopha.2023.115837

[33] Li, Z.-M., Xu, S.-W. & Liu, P.-Q. Salvia miltiorrhizaBurge (Danshen): a golden herbal medicine in cardiovascular therapeutics. *Acta Pharm. Sin.* **39**, 802–824 (2018). 10.1038/aps.2017.193 PMC5943903 29698387

[34] Xu, W. et al. Salvianolic acid B exerts an anti-hepatocellular carcinoma effect by regulating the Hippo/YAP pathway and promoting pSmad3L to pSmad3C simultaneously. *Eur. J. Pharm.* **939**, 175423 (2023). 10.1016/j.ejphar.2022.175423 36509132

[35] Zhao, Y. et al. Salvianolic acid B inhibits atherosclerosis and TNF-α-induced inflammation by regulating NF-κB/NLRP3 signaling pathway. *Phytomedicine* **119**, 155002 (2023). 37572566 10.1016/j.phymed.2023.155002

[36] Lopez-Ramirez, M. A. et al. Built-in active microneedle patch with enhanced autonomous drug delivery. *Adv. Mater.* **32**, e1905740 (2020). 31682039 10.1002/adma.201905740 PMC7014935

[37] Pandey, S., Kant, S., Khawary, M. & Tripathi, D. Macrophages in microbial pathogenesis: commonalities of defense evasion mechanisms. *Infect. Immun.* **90**, e0029121 (2022). 34780281 10.1128/iai.00291-21 PMC9119111

[38] Zheng, M. & Kanneganti, T.-D. The regulation of the ZBP1–NLRP3 inflammasome and its implications in pyroptosis, apoptosis, and necroptosis (PANoptosis). *Immunol. Rev.* **297**, 26–38 (2020). 32729116 10.1111/imr.12909 PMC7811275

[39] Yamaguchi, T. et al. Oxidative stress inhibits endotoxin tolerance and may affect periodontitis. *J. Dent. Res* **102**, 331–339 (2023). 36529984 10.1177/00220345221138523

[40] Lv, X., Fan, C., Jiang, Z., Wang, W., Qiu, X. & Ji, Q. Isoliquiritigenin alleviates *P. gingivalis*-LPS/ATP-induced pyroptosis by inhibiting NF-κB/ NLRP3/GSDMD signals in human gingival fibroblasts. *Int. Immunopharmacol.* **101**, 108338 (2021). 34794890 10.1016/j.intimp.2021.108338

[41] Riley, J. S. & Tait, S. W. Mitochondrial DNA in inflammation and immunity. *EMBO Rep.* **21**, e49799 (2020). 32202065 10.15252/embr.201949799 PMC7132203

[42] Decout, A., Katz, J. D., Venkatraman, S. & Ablasser, A. The cGAS–STING pathway as a therapeutic target in inflammatory diseases. *Nat. Rev. Immunol.* **21**, 548–569 (2021). 33833439 10.1038/s41577-021-00524-z PMC8029610

[43] Xia, X. et al. Inhibiting mtDNA–STING–NLRP3/IL-1β axis-mediated neutrophil infiltration protects neurons in Alzheimer’s disease. *Cell Prolif.* **57**, e13529 (2024). 37528567 10.1111/cpr.13529 PMC10771109

[44] Sundaram, B. et al. NLRP12–PANoptosome activates PANoptosis and pathology in response to heme and PAMPs. *Cell* **186**, 2783–2801.e20 (2023). 37267949 10.1016/j.cell.2023.05.005 PMC10330523

[45] Kotkowska, A., Sewerynek, E., Domańska, D., Pastuszak-Lewandoska, D. & Brzeziańska, E. Single nucleotide polymorphisms in the STAT3 gene influence AITD susceptibility, thyroid autoantibody levels, and IL6 and IL17 secretion. *Cell Mol. Biol. Lett.* **20**, 88–101 (2015). 26204395 10.1515/cmble-2015-0004

[46] Kumari, S., Dhapola, R. & Reddy, D. H. Apoptosis in Alzheimer’s disease: insight into the signaling pathways and therapeutic avenues. *Apoptosis* **28**, 943–957 (2023). 37186274 10.1007/s10495-023-01848-y

[47] Ottani, A. et al. Modulation of the JAK/ERK/STAT signaling in melanocortin-induced inhibition of local and systemic responses to myocardial ischemia/reperfusion. *Pharm. Res* **72**, 1–8 (2013). 10.1016/j.phrs.2013.03.005 23535516

[48] Xiao, Z. et al. Pharmacological effects of salvianolic acid B against oxidative damage. *Front. Pharm.* **11**, 572373 (2020). 10.3389/fphar.2020.572373 PMC7741185 33343348

[49] Zheng, X., Liu, H., Ma, M., Ji, J., Zhu, F. & Sun, L. Anti-thrombotic activity of phenolic acids obtained from *Salvia miltiorrhiza* f. alba in TNF-α-stimulated endothelial cells via the NF-κB/JNK/p38 MAPK signaling pathway. *Arch. Pharm. Res* **44**, 427–438 (2021). 33847919 10.1007/s12272-021-01325-7

[50] Li, J. et al. Omics and transgenic analyses reveal that salvianolic acid B exhibits its anti-inflammatory effects through inhibiting the Mincle-Syk-related pathway in macrophages. *J. Proteome Res.* **20**, 3734–3748 (2021). 34080425 10.1021/acs.jproteome.1c00325

[51] Wang, Y.-L. et al. Screening of the ubiquitin-proteasome system activators for anti-Alzheimer’s disease by the high-content fluorescence imaging system. *Chin. J. Nat. Med* **20**, 33–42 (2022). 35101248 10.1016/S1875-5364(22)60152-3

[52] Hu, S. et al. Salvianolic acid B alleviates liver injury by regulating lactate-mediated histone lactylation in macrophages. *Molecules* **29**, 236 (2024). 38202819 10.3390/molecules29010236 PMC10780734

[53] Sun, J.-M. et al. Salvianolic acid B protects against UVB-induced skin aging via activation of NRF2. *Phytomedicine* **130**, 155676 (2024). 38820663 10.1016/j.phymed.2024.155676

