# Supplementary material for: Porphyromonas gingivalis induces Zbp1-mediated macrophages PANoptosis in periodonitis pathophysiology
Source: Exp Mol Med. 2025 May 1;57(5):964–78. doi: 10.1038/s12276-025-01443-y (PMC12130536; doi:10.1038/s12276-025-01443-y)
Supplement: Supplementary file 1 — Supplementary Information [file 12276_2025_1443_MOESM1_ESM.pdf]

Supplementary Information

Porphyromonas gingivalis induces Zbp1-mediated macrophages PANoptosis in periodonitis pathophysiology

Jin Wu[1,2*], Zixiang Guo[1,2*], Long Wang[1,2], Yue Shen[1,2], Xiang Li[1,3], Zhewei Zhang[1,2], Xiao Han[1,2], Jianlan Zhang[2], Kunzhan Cai[1,2✉] and Chunbo Tang[1,2✉]

[1] State Key Laboratory Cultivation Base of Research, Prevention and Treatment for Oral Diseases, Nanjing Medical University, 140 Hanzhong Road, Nanjing 210029, Jiangsu Province, China

[2] Department of Oral Implantology Affiliated Hospital of Stomatology, Nanjing Medical University, 136 Hanzhong Road, Nanjing 210029, Jiangsu Province, China

[3] Department of Oral and Maxillofacial Surgery Affiliated Hospital of Stomatology, Nanjing Medical University, 136 Hanzhong Road, Nanjing 210029, Jiangsu Province, China

[*]These authors contributed equally to this article: Jin Wu, Zixiang Guo.

[✉]**Corresponding Authors:**

Kunzhan Cai

Department of Oral Implantology Affiliated Hospital of Stomatology, Nanjing Medical University, 136 Hanzhong Road, Nanjing 210029, Jiangsu Province, China

Tel: +86-69593083

E-mail: caikunzhan@njmu.edu.cn

Chunbo Tang

Department of Oral Implantology Affiliated Hospital of Stomatology, Nanjing Medical University, 136 Hanzhong Road, Nanjing 210029, Jiangsu Province, China

Tel: +86-13813829999

E-mail: cbtang@njmu.edu.cn

Supplemental Figures

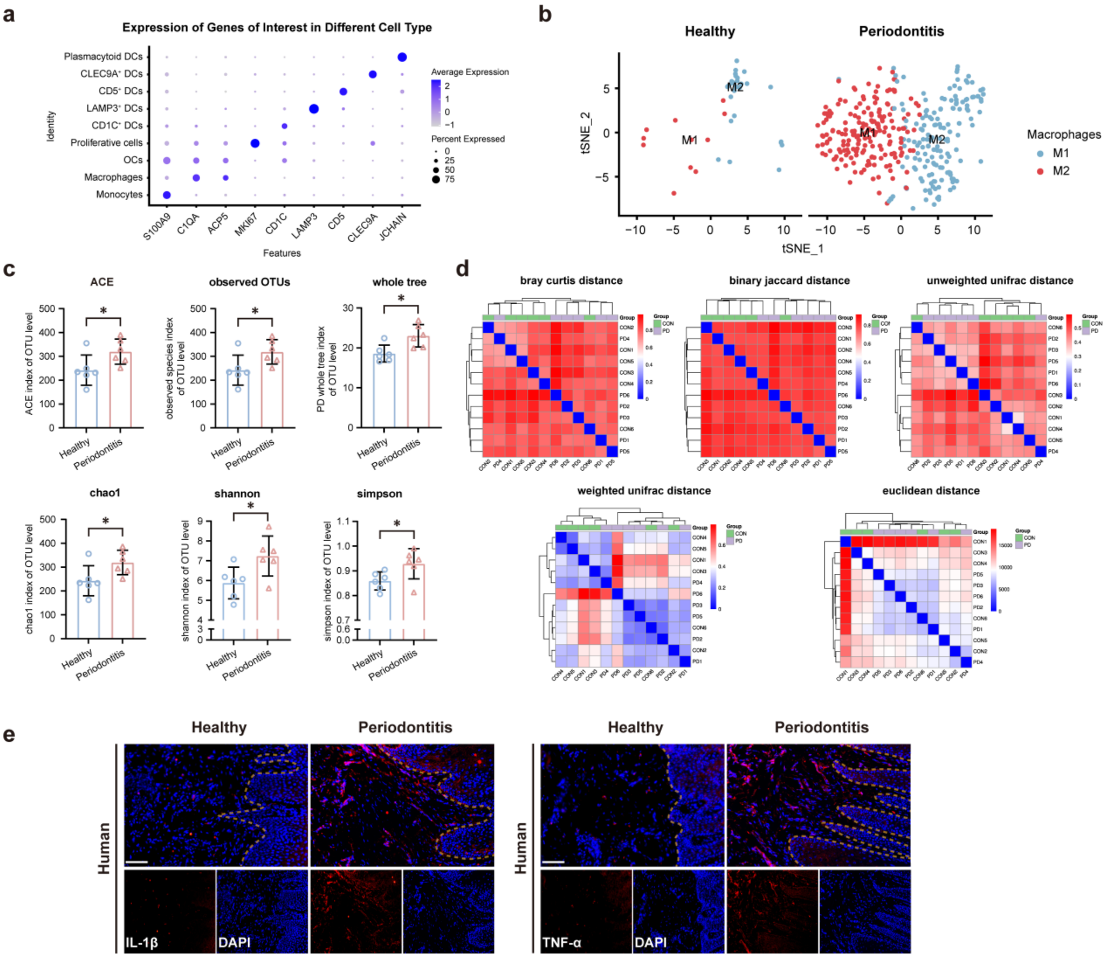

Supplementary Fig. 1 *P. gingivalis* infection triggered macrophage infiltration and induced apoptosis and necrosis in periodontal tissues. (a) Dot plots of marker gene expression (Z-scaled) and expressed cell percentages (dot size) in major cell subtypes of monocyte clusters. **(b)** TSNE plot representing the M1 and M2 macrophage clusters of cells identified across healthy and periodontitis. **(c)** Alpha diversity showing increased microbial richness in periodontitis. **(d)** Beta diversity revealing distinct community compositions between samples. **(e)** Heightened IL-1β and TNF-α expression in periodontitis tissues. n=5. Scale bar, 50 μm. All data were derived from independent experiments. *$p < 0.05$.

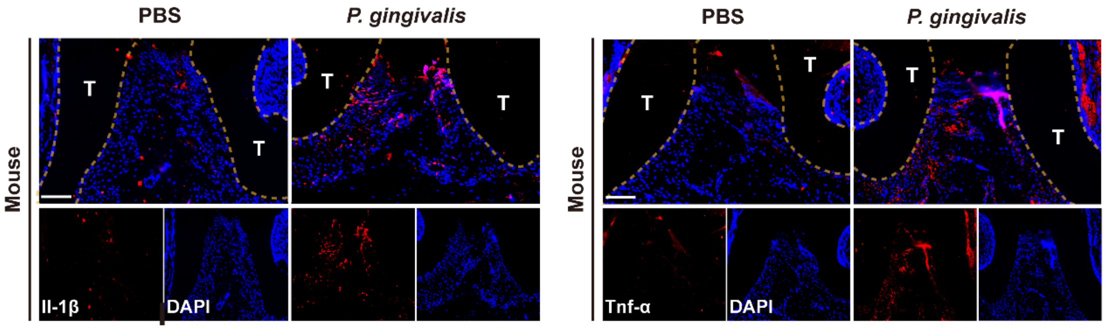

Supplementary Fig. 2 Upregulated IL-1β and TNF-α in *P. gingivalis*-infected periodontitis tissues. *n*=5. Scale bar, 50 μm. All data were derived from independent experiments.

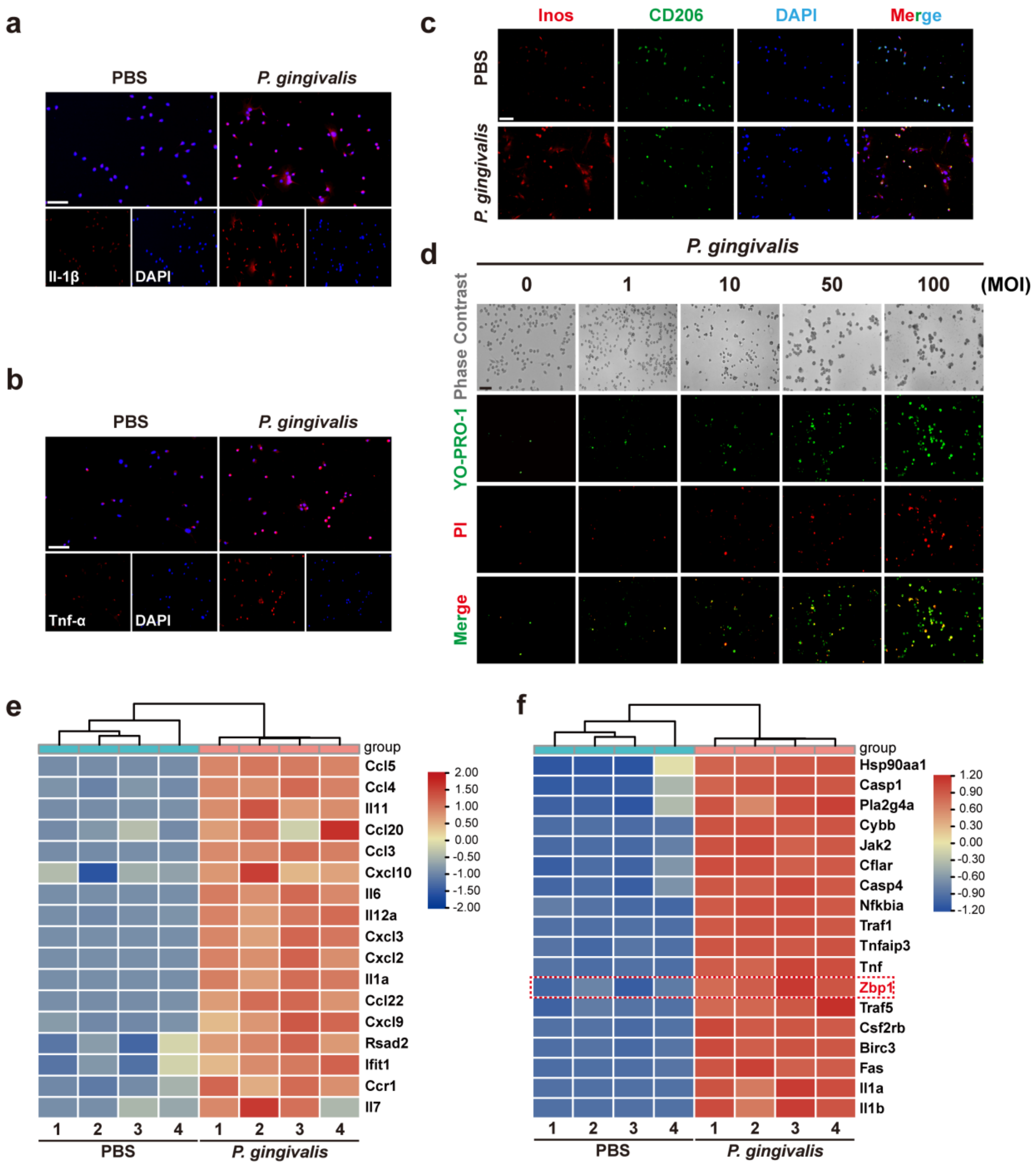

Supplementary Fig. 3 *P. gingivalis* infection activated Zbp1 in BMDMs. (a-c) Increased IL-1β, TNF-α expression, and enhanced M1 polarization in BMDMs co-cultured with *P. gingivalis*. *n=3*. Scale bar, 50 µm. **(d)** Enhanced apoptosis and necrosis in BMDMs after *P. gingivalis* stimulation. *n=3*. Scale bar, 50 µm. **(e)** Heat map displaying the dysregulation of inflammatory factors and immune response-related markers in *P. gingivalis*-infected BMDMs. *n*=4. **(f)** Heat map depicting upregulated cell death-related DEGs after *P. gingivalis* infection. *n*=4. All data were derived from independent experiments.

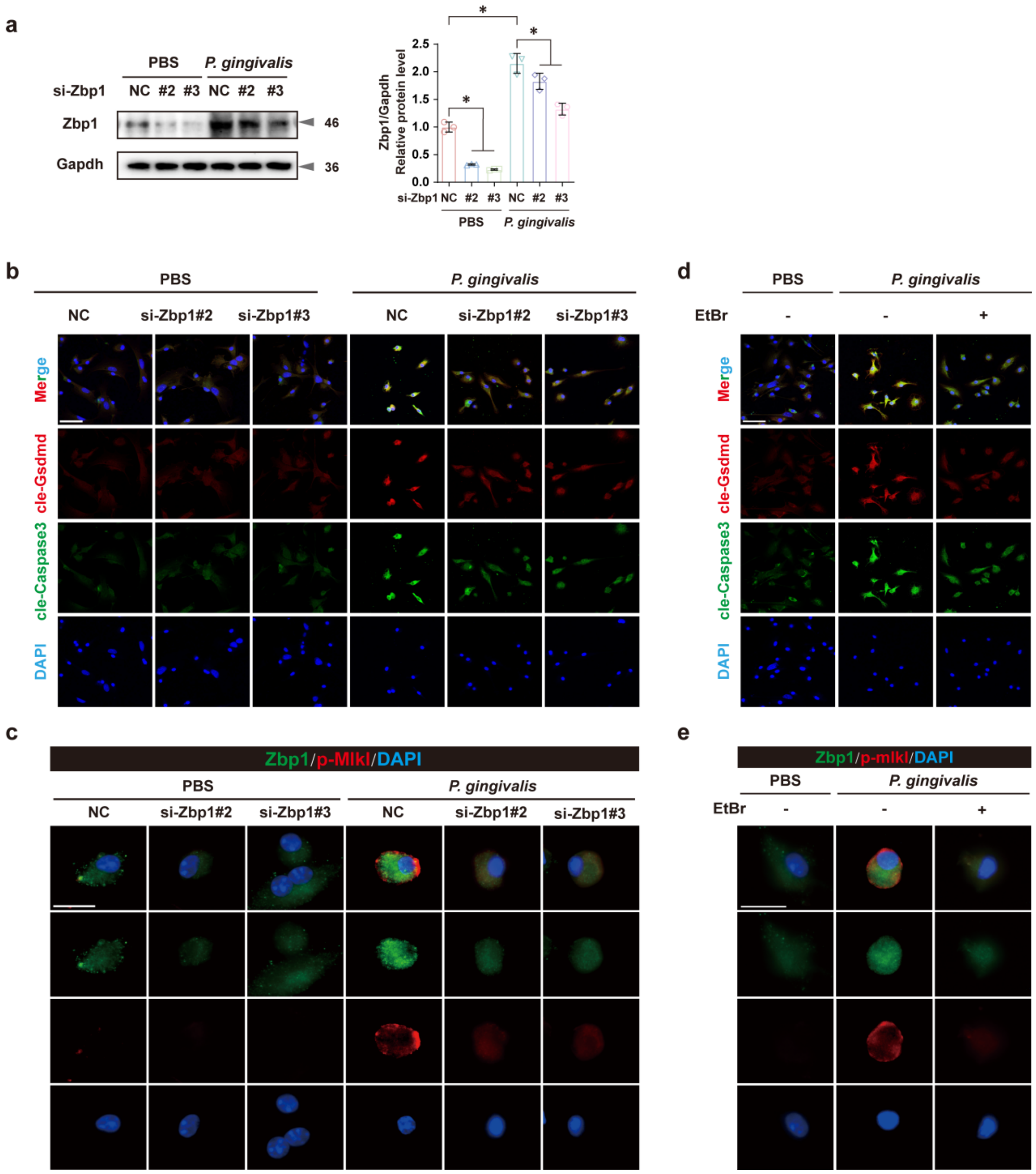

Supplementary Fig. 4 Targeted deletion of Zbp1 in BMDMs protected against PANoptosis induced by *P. gingivalis* infection. (a) Zbp1 knockdown significantly reduced the *P. gingivalis*-induced upregulation of Zbp1 levels. *n=3.* **(b)** Reduced cleaved Gsdmd and cleaved Caspase3 expression with Zbp1 siRNAs treatment. *n=3.* Scale bar, 50 μm. **(c)** IF images of p-Mlkl localization on the plasma membrane following *P. gingivalis* stimulation. *n=3.* Scale bar, 10 μm. IF staining showing suppressed expression of **(d)** cleaved Gsdmd, cleaved Caspase3 (Scale bar, 50 μm), and **(e)** p-Mlkl (Scale bar, 10 μm), with EtBr treatment. *n*=3. All data were derived from independent experiments. $*p < 0.05$.

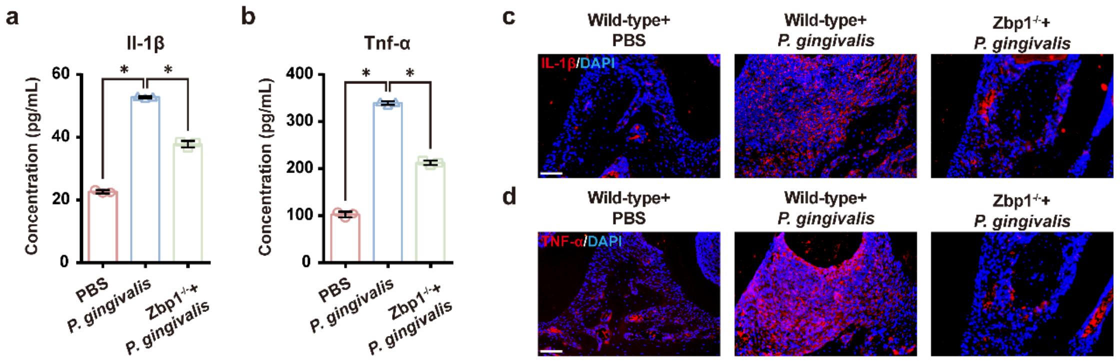

Supplementary Fig. 5 Deletion of Zbp1 protected against PANoptosis activation and periodontal tissue destruction in *P. gingivalis*-induced mouse periodontitis. **(a-b)** Levels of IL-1β and TNF-α decreased markedly in BMDMs derived from Zbp1$^{-/-}$ mice post-*P. gingivalis* infection. n=3. **(c-d)** IF images revealing decreased IL-1β and TNF-α expression in periodontitis lesions of Zbp1$^{-/-}$ mice. n=5. Scale bar, 50 µm. All data were derived from independent experiments. $*p < 0.05$.

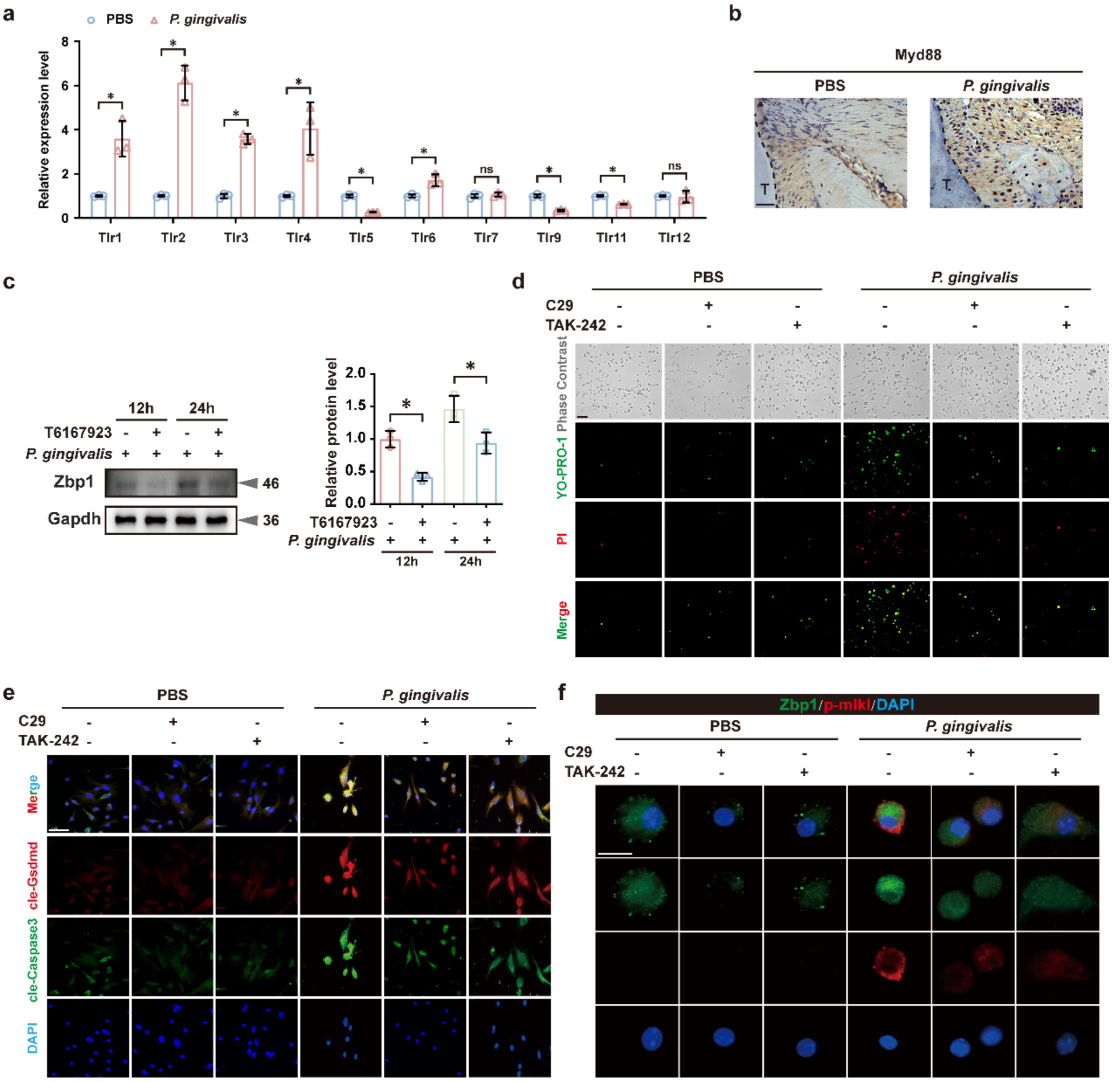

Supplementary Fig. 6 *P. gingivalis* activated Zbp1 in BMDMs via the Tlr2/4-JNK pathways. (a) qRT-PCR analysis of Tlr gene expression profiles in *P. gingivalis*-stimulated BMDMs. *n*=3. **(b)** IHC assay showing increased expression of Myd88 in *P. gingivalis*-induced periodontitis. *n*=5. Scale bar, 50 μm. **(c)** Reduced Zbp1 expression with Myd88 inhibitor in the infected BMDMs. *n*=3. **(d)** Suppressed apoptosis and necrosis in *P. gingivalis*-stimulated BMDMs following C29 and TAK-242 treatment. *n*=3. Scale bar 50 μm. **(e-f)** Decreased expression of cleaved Gsdmd, cleaved Caspase3, and p-Mlkl in *P. gingivalis*-stimulated BMDMs treated with C29 and TAK-242. *n*=3. Scale bar (e: 50 μm, f: 100 μm). All data were derived from independent experiments. ns no significance, **p* < 0.05.

a

	Matrix ID	Name	Score	Relative score	Sequence ID	Start	End	Strand	Predicted sequence
1	MA0147.2	MA0147.2.Myc	15.200638	0.9979261657264286	seq1	1499	1508	-	CCATGTGGTT
2	MA0079.2	MA0079.2.SP1	14.453586	0.9952989230924939	seq1	1073	1082	+	CCCCTCCCCC
3	MA0144.1	MA0144.1.Stat3	13.847042	0.9425356507729611	seq1	1839	1848	-	TGCCTGGAAG
4	MA0143.1	MA0143.1.Sox2	13.819387	0.8738960799048621	seq1	1716	1730	-	CTATTGTTATGAAAC
5	MA0519.1	MA0519.1.Stat5	13.079105	0.9536135797653155	seq1	1114	1124	+	TCTTCTAAGAA

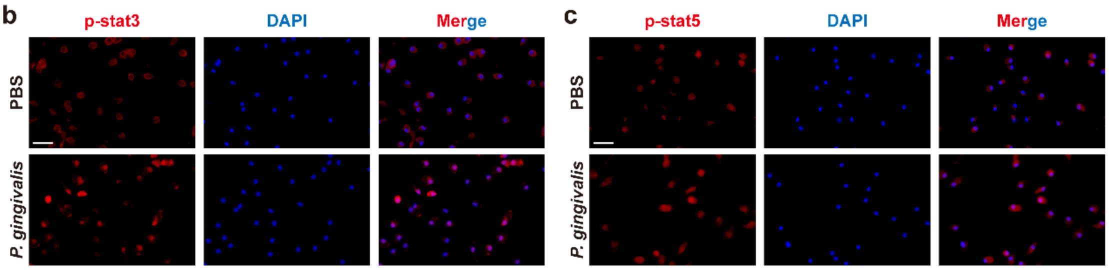

Supplementary Fig. 7 *P. gingivalis* activated TFs Stat3 and Stat5 to promote Zbp1 transcription. (a) Predicted scores suggesting potential TFs involved in Zbp1 transcription. **(b-c)** Nuclear translocation of Stat3 and Stat5 in BMDMs post-*P. gingivalis* infection. n=3. Scale bar, 50 µm. All data were derived from independent experiments. *$p < 0.05$.

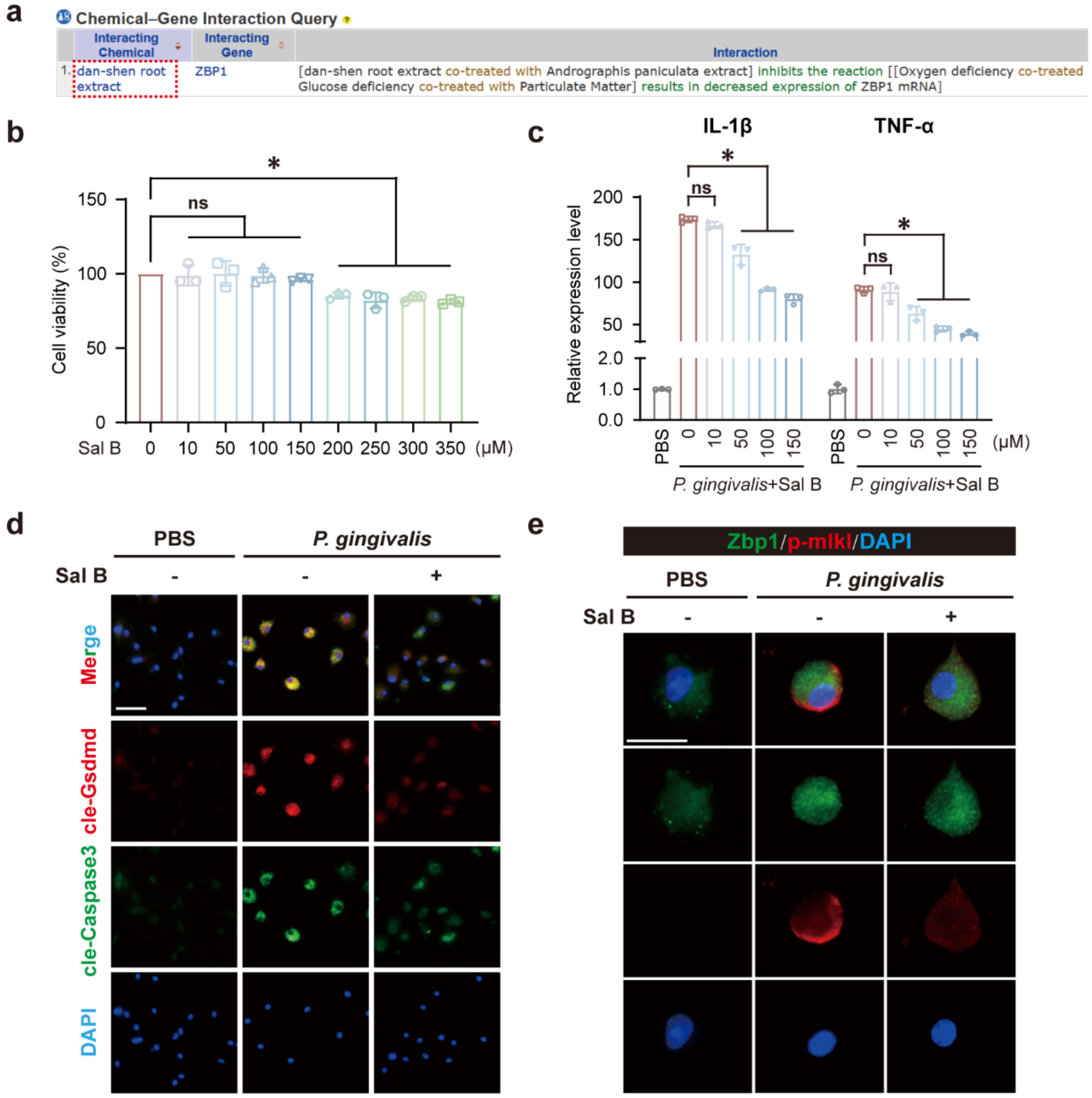

Supplementary Fig. 8 MNs delivery of Sal B alleviated *P. gingivalis*-induced periodontal resorption and promoted mucosal wound healing. (a) Prediction of Danshen extract interaction with Zbp1 using the CTD database. **(b)** CCK-8 assay evaluating the impact of Sal B on cell viability. *n*=3. **(c)** qRT-PCR showing reduced IL-1β and TNF-α levels in *P. gingivalis*-infected cells treated with Sal B (≥ 50 μM) *n*=3. **(d-e)** IF staining revealing significant suppression of cleaved Gsdmd, cleaved Caspase3, and p-Mlkl upon Sal B treatment in *P. gingivalis*-infected cells. *n*=3. Scale bar (d: 50 μm, e: 100 μm). All data were derived from independent experiments. ns no significance, *$p < 0.05$.

Supplementary Tables

Supplementary Table 1. Clinical characteristics of the patients obtaining subgingival plaque samples

	Healthy					Periodontitis					
**No.**	**1**	**2**	**3**	**4**	**5**	**1**	**2**	**3**	**4**	**5**	**6**
Age	65	44	57	45	43	48	40	69	46	54	49
Gender	female	male	male	female	female	male	female	female	male	male	male
Probing depth (mm)	2	3	1	3	4	10	6	9	8	10	9
Clinical attachment loss (mm)	1	0	0	1	2	5	4	5	6	7	5
Bleeding on probing index	0	0	1	1	1	2	2	4	3	4	4

Supplementary Table 2. Clinical characteristics of the patients obtaining gingival specimens

	Healthy					Periodontitis				
**No.**	**1**	**2**	**3**	**4**	**5**	**1**	**2**	**3**	**4**	**5**
Age	57	43	56	48	47	43	56	41	44	54
Gender	male	female	male	male	female	male	male	male	female	female
Probing depth (mm)	3	4	2	4	3	8	7	8	8	9
Clinical attachment loss (mm)	1	1	0	1	0	7	10	5	11	6
Bleeding on probing index	1	1	0	1	0	3	4	5	5	4

Supplementary Table 3. Primer sequences used in the experiments

	Forward Sequence (5'-3')	Reverse Sequence (5'-3')
**qRT-PCR**		
Gapdh	AGGTCGGTGTGAACGGATTTG	GGGGTCGTTGATGGCAACA
Hsp90aa1	GACGCTCTGGATAAAATCCGTT	TGGGAATGAGATTGATGTGCAG
Casp1	ACAAGGCACGGGACCTATG	TCCCAGTCAGTCCTGGAAATG
Pla2g4a	CAGCACATTATAGTGGAACACCA	GTCCAGCATATCGCCAAAGGT
Cybb	AGTGCGTGTTGCTCGACAA	GCGGTGTGCAGTGCTATCAT
Jak2	GGAATGGCCTGCCTTACAATG	TGGCTCTATCTGCTTCACAGAAT
Cflar	GGTGGAAGAGTGTCTTGATGAAG	CCCTGACGTTAGGTGCAGC
Casp4	AGCGTTGGGTTTTTGTAGATGC	CCTTGTGAACTCTTCAGGGGA
Nfkbia	TGAAGGACGAGGAGTACGAGC	TGCAGGAACGAGTCTCCGT
Traf1	AGGGTGGTGGAATTACAGCAA	GCAGTGTAGAAAGCTGGAGAG
Tnfaip3	ACCATGCACCGATACACGC	AGCCACGAGCTTCCTGACT
Tnf	CAGGCGGTGCCTATGTCTC	CGATCACCCCGAAGTTCAGTAG
Zbp1	AAGAGTCCCCTGCGATTATTTG	TCTGGATGGCGTTTGAATTGG
Traf5	CACTCCGTGCTTCACAACC	GCGTTTTTGCAGTAGACGTGTA
Csf2rb	ACAGAGAACCTAGATCGAGCC	GTGTACTCTTCGCTCCACTTG
Bir3	TGAAGAGTGCTGACACCTTTG	GGAAAAGCTGAATACGTGGACAA
Fas	GCGGGTTCGTGAAACTGATAA	GCAAAATGGGCCTCCTTGATA
Il1b	GAAATGCCACCTTTTGACAGTG	TGGATGCTCTCATCAGGACAG
Il1a	TCTATGATGCAAGCTATGGCTCA	CGGCTCTCCTTGAAGGTGA
Cyt B	ATGACCCACCAATCACATGC	CTTCTCCCTTGGAGGAGTAGG
Nd2	ATGGCTGAGACGCTGAGAAG	TGGGAGCTCTTTGGTGAGTA
**ChIP**		
(Stat3)-Zbp1-55F: GACAACTGAAGCTACTCCCC		
(Stat3)-Zbp1-243R: TGTTGTCGGTCAACCCAGGT		
(Stat5)-Zbp1-770F: ATGGTGGGGATCACAACGAC		
(Stat5)-Zbp1-953R: CCTTTCTTCAGCATTTTCCTC		

Supplementary Table 4. siRNA sequences used in the experiments

siRNAs	Senquence (5'-3')
si-Zbp1#1	CCCTCAATCAAGTCCTTTA
si-Zbp1#2	GCCTGCAACATGGAGCATA
si-Zbp1#3	CCTGTATTCCATGAGAAAT
si-Tlr2	GGAAGGAGCTGGAAGGATA
si-Tlr4	GGAGACCTGTGGGTTATGA
si-Myd88	GGAAGAACCTGTGGAAATA

Supplemental Materials and Methods

Immunofluorescence (IF)

For IF staining of periodontal tissue samples, a heat-induced antigen retrieval process was initiated by immersing sections in an antigen repair buffer (pH = 8.0) containing EDTA at 100°C for 20 min. Subsequently, the staining of cell samples and tissue sections followed similar procedures. They were permeabilized with 0.1% Triton X-100 and treated with 3% hydrogen peroxide for 20 min. Following blocking with 5% bovine serum albumin (BSA) for 1 h, samples were incubated with primary antibodies (anti-CD68, 66231-2-Ig, Proteintech, 1:3000; anti-p-MLKL, #37333, CST, 1:1600; anti-F4/80, #71299, CST, 1:100; anti-Zbp1, #DF14090, Affinity, 1:200; anti-cle-Gsdmd, #36425, CST, 1:500; anti-cle-Caspase3, #9661, CST, 1:400; anti-p-Stat3, ab76315, Abcam, 1:500; anti-p-Stat5, ab98338, Abcam, 5 μg/mL) overnight at 4°C, followed by incubation with fluorophore-conjugated secondary antibodies (Abbkine, Wuhan, China) for 1 h. Finally, coverslips with antiquenching solution (containing DAPI) were mounted on the samples of cells or periodontal sections. Samples were observed and photographed under a confocal laser scanning microscopy (CLSM, Zeiss, Germany), and the percentages of macrophages ($CD68^+$ or $F4/80^+$) and necrotic cells ($p\text{-}MLKL^+$) were calculated using the ImageJ software 1.8.0 (National Institutes of Health, Bethesda, Maryland, USA).

Terminal deoxynucleotidyl transferase dUTP nick end labeling (TUNEL)

TUNEL assay was performed according to the manufacturer’s instructions (Vazyme, China). Briefly, the paraffin sections were deparaffinized and permeabilized with proteinase K for 30 min, followed by permeabilization with 0.1% Triton-X100. Subsequently, the TUNEL reaction mixture was incubated with the sections for 1 h at 37°C, with total nuclei stained using DAPI. The percentage of apoptotic cells (TUNEL-positive) was calculated using the ImageJ software.

Micro-computed tomography (micro-CT) analysis

The microarchitectural properties of the maxillary bones were scanned using a micro-CT system (Micro CT50, Scanco Medical AG, Switzerland). The scanning parameters were configured at a voltage of 55 kVp, a current of 70 μA, and a pixel size of 15.6 μm. Subsequently, three-dimensional reconstruction of the regions of interest, specifically focusing on the mesial and distal alveolar bones of the second maxillary molar teeth, was executed using CTvox software (version 3.3.0, Burker MicroCT NV, Germany).

Hematoxylin and Eosin (HE), Tartrate-Resistant Acid Phosphatase (TRAP), and Immunohistochemistry (IHC) staining

For HE staining, sections were immersed in hematoxylin for 5 min, followed by differentiation in an acid ethanol solution. Subsequently, bluing was achieved via immersion in saturated lithium carbonate. The final step involved incubation with eosin for 3 min. Sections were stained using a TRAP kit (Jiancheng, Nanjing, China) according to the manufacturer's instructions. Mature osteoclasts were identified based on the presence of three or more positively stained nuclei. For IHC staining, the sections were subjected to a 1-hour incubation in BSA. Subsequently, primary antibodies (anti-Zbp1, 13285-1-AP, Proteintech, 1:200; anti-Tlr2, 17236-1-AP, Proteintech, 1:200; anti-Tlr4, 66350-1-Ig, Proteintech, 1:200; anti-Myd88, 67969-1-Ig, Proteintech, 1:250) were used to probe the tissue sections overnight at 4°C. The sections were then incubated with secondary antibodies (Abbkine) for 20 min. The expression of the target protein was detected using a DAB substrate (MXB, China), and hematoxylin was employed for nuclear staining.

Antibiotics treatment

A combination of amoxicillin (1.5 mg/mL) and metronidazole (0.5 mg/mL) was dissolved in the drinking water provided to the mice and administered continuously for 10 days, aiming to broadly eliminate both aerobic and anaerobic bacteria in the oral cavity. To evaluate the bacterial load after treatment, mouse saliva samples were collected using oral swabs following a 2-hour fasting period. A sterile swab was

rotated 5 times (for a total of 10 seconds) within the mouse's oral cavity, with efforts made to maintain consistent sampling pressure. After collection, the swab was immersed in 1 mL of PBS for sample extraction. Subsequently, 100 μL of the extracted sample was cultured on LB agar plates.

YO-PRO-1 and Propidium Iodide (PI) dual staining assay

The Apoptosis and Necrosis Detection Kit with YO-PRO-1 and PI was purchased from Beyotime (China). BMDMs were seeded in a 12-well plate at a density of 25×10^4 cells per well and subsequently infected with *P. gingivalis* at a multiplicity of infection (MOI) of 100 for 24 h. After removing the culture medium, the BMDMs were washed thrice with PBS. Subsequently, 500 μL of YO-PRO-1/PI detection working solution was added to each well, and the cells were incubated at 37°C in the dark for 20 min. Following the incubation period, fluorescence was observed using a fluorescence microscope (Leica, Germany), where YO-PRO-1-positive cells exhibited green fluorescence, and PI-positive cells exhibited red fluorescence.

Quantitative real-time polymerase chain reaction (qRT-PCR)

Total RNA isolation from cultured cells was conducted using TRIzol reagent (Invitrogen, USA), followed by cDNA synthesis with Primescript RT Reagent (Vazyme). Subsequent qRT-PCR analysis was performed with the SYBR Premix Ex Taq (Vazyme) on the QuantStudio Q7 Real-time PCR system (Thermo Fisher Scientific, USA). Following the normalization of target gene expression to Gapdh levels, data quantification was performed utilizing the $2^{-\Delta\Delta Ct}$ method. The primer sequences utilized in this study are documented in Table S3.

Western Blot

After being infected with *P. gingivalis* at a MOI of 100 for 24 h, bone marrow-derived macrophages (BMDMs) were lysed using radio immunoprecipitation assay (RIPA) lysis buffer (Beyotime) containing phenyl methane sulfonyl fluoride (PMSF, Beyotime). Following centrifugation of the homogenate for 15 min at 4℃ (12,000 ×g),

the supernatants were collected and separated through sodium dodecyl sulfate-polyacrylamide gel electrophoresis (SDS-PAGE). The membranes were blocked with 5% BSA (NCM, China) for 2 h and subsequently incubated with primary antibodies: Zbp1 (1:1000, 13285-1-AP, Proteintech, China); Gapdh (1:50000, 60004-1-Ig, Proteintech, China); p-Ripk3 (#91702, 1:1000, CST, USA); Ripk3 (17563-1-AP, 1:1000, Proteintech, China); cle-Caspase8 (#9429, 1:1000, CST, USA); Caspase8 (#4927, 1:1000, CST, USA); cle-Caspase3 (#9661, 1:1000, CST, USA); Caspase3 (#9662, 1:1000, CST, USA); Nlrp3 (#15101, 1:1000, CST, USA); cle-Gsdmd (#36425, 1:1000, CST, USA); Gsdmd (#39754, 1:1000, CST, USA); p-Mlkl (#37333, 1:1000, CST, USA); Mlkl (66675-1-Ig, 1:5000, Proteintech, China); p-p65 (82335-1-RR, 1:2000, Proteintech, China); p65 (80979-1-RR, 1:5000, Proteintech, China); p-ERK (80031-1-RR, 1:2000, Proteintech, China); ERK (11257-1-AP, 1:2000, Proteintech, China); p-JNK (80024-1-RR, 1:1000, Proteintech, China); JNK (66210-1-Ig, 1:3000, Proteintech, China); p-p38 (28796-1-AP, 1:1000, Proteintech, China); p38 (14064-1-AP, 1:2000, Proteintech, China); p-Stat3 (#9145, 1:2000, CST, USA); Stat3 (10253-2-AP, 1:2000, Proteintech, China); p-Stat5 (28951-1-AP, 1:1000, Proteintech, China); Stat5 (13179-1-AP, 1:500, Proteintech, China) at 4℃ overnight. The following day, membranes were incubated with secondary antibodies (Abbkine, USA) for 1 h after being washed thrice with Tris-buffered saline-Tween 20 (TBST). Chemiluminescence detection was performed using a Western ECL Substrate kit (Abbkine, USA), and quantitative analysis was carried out using the ImageJ software.

Enzyme-linked immunosorbent assay (ELISA)

ELISA assay was performed to detect the release of pro-inflammatory cytokines. BMDMs were seeded in a 12-well plate a density of 25×10^4 cells per well and subsequently incubated with PBS or *P. gingivalis* (MOI=100) at 37°C for 24 h. Then, the culture solution was gathered for the quantification of proinflammatory cytokines IL-1β and TNF-α derived from BMDMs, using ELISA kits (KE10003 and KE10002, Proteintech, China) in accordance with the manufacturer's protocol. Standard curves

were employed to calculate the concentrations of cytokines. Subsequently, absorbance OD values were measured using a microplate reader at a wavelength of 450 nm. The standard curve was plotted using ELISACalc software, with the concentration of the standard on the horizontal axis and the absorbance OD value on the vertical axis, thereby enabling the accurate determination of cytokine concentrations.

Lactate dehydrogenase (LDH) release

Under physiological conditions, LDH is sequestered within the cytoplasm and incapable of traversing the cell membrane. However, in instances where a cell undergoes damage or death, leading to the disruption of cell membrane integrity, LDH is released into the extracellular space. Following treatment with *P. gingivalis* at a MOI of 100 for 24 h, LDH Cytotoxicity Assay Kit (Beyotime) was used to assess the level of LDH release in BMDMs, thereby detecting cellular injury and death.

Small interfering RNAs (siRNAs) transfection

Before transfection, BMDMs were seeded into 6-well plates at a density of 5×10^5 cells per well and allowed to adhere for 12 h. The siRNAs and corresponding control (si-NC) were purchased from RiboBio. Following the instructions provided by the manufacturer, transfection complexes were prepared with 5 μL Lipofectamine 3000 (Thermo Fisher Scientific) and 10 μL of siRNA (25 nM) in 500 μL DMEM medium. After transfection for 6 h, the medium was replaced with fresh DMEM, and the cells were cultured further to await subsequent treatment and assays. The sequences of siRNAs used in this study are listed in Supplementary Table 4.

\

Mitochondrial membrane potential (ΔψM) assay

The ΔψM assay was conducted employing the JC-1 mitochondrial membrane potential detection kit (Beyotime). Briefly, cells were incubated with JC-1 staining solution (2.0 μg/mL) for 20 min at 37 ℃, followed by washing three times with PBS and examination under a fluorescence microscope (Leica, Germany). The relative fluorescence units (RFU) value was determined by the ratio of red JC-1 aggregates

fluorescence to green JC-1 monomers fluorescence.

Reactive oxygen species (ROS), mitochondrial ROS (mtROS) and mitochondrial fragmentation detection

The DCFH-DA probe (Jiancheng, Nanjing, China) was diluted to a concentration of 10 μM using serum-free DMEM. Cells were incubated with DCFH-DA solution for 20 min at 37 ℃, followed by thorough rinsing with PBS and examination under a fluorescence microscope (Leica).

For the detection of mtROS, cells were treated with the Mito-SOX Red probe (Invitrogen, USA), involving a 10-minute incubation of cells with 2 μM fluorochrome. Subsequently, cells were washed three times with PBS, followed by examination using a fluorescence microscope (Leica).

Mito-Tracker Deep Red FM stock solution (200 μM, Beyotime) was diluted at a 1:1000 ratio in DMEM to prepare the working solution. Then, cells were cultured with the working solution and incubated at 37°C for 30 minutes. Mitochondrial morphology was observed using a confocal laser scanning microscopy (Zeiss), and mitochondrial fragmentation levels were quantified via ImageJ software.

Adenosine triphosphate measurement (ATP)

ATP concentration was determined using an ATP assay kit (Beyotime). BMDMs were seeded in a 6-well plate at a density of 5×10^5 cells per well and subsequently infected with *P. gingivalis* at a MOI of 100. After a 24-hour incubation period, the culture medium was removed, and 200 μL of lysis solution was added to each well on a 6-well plate. The total protein was isolated by centrifugation at 12,000 ×g for 5 min. Following this, 100 μL of the supernatant was mixed with 100 μL of ATP detection solution. The relative light units (RLU) were measured using a luminescence microplate reader (Bio Tek, USA), and the concentration of ATP in the sample was calculated based on the standard curve.

Dual-luciferase reporter assay

The pGL3 Basic Zbp1_Pro vector was generated by constructing a Zbp1 promoter truncation sequence upstream of Luc in the pGL3 Basic vector. Subsequently, 293T cells (1×10^5) were seeded in a six-well plate and cultured for 24 h. The cells were then co-transfected with the pGL3 basic vector, pGL3 Basic Zbp1_Pro vector, and Stat3 or Stat5 plasmid using Lipofectamine 3000. Relative luciferase activity (firefly luciferase activity/Renilla luciferase activity) was measured 48 h after transfection using a Dual-Luciferase Reporter Assay Kit (GeneCopoeia, USA).

Cell viability assay

To assess the viability of BMDMs exposed to Salvianolic acid B (Sal B), a preliminary investigation was conducted using a concentration gradient of Sal B (0, 10, 50, 100, 150, 200, 250, 300, and 350 μM). Following a 24-hour incubation period, the culture medium was replaced with 10% Cell Counting Kit-8 (CCK-8) solution (α-MEM: CCK-8 = 9: 1) and incubated in a 37°C incubator in the dark for 2 h. The absorbance of the solution was measured at 450 nm using a microplate reader.
